# Overview on the Thermally Activated Delayed Fluorescence and Mechanochromic Materials: Bridging Efficiency and Versatility in LECs and OLEDs

**DOI:** 10.3390/ma18122714

**Published:** 2025-06-09

**Authors:** Raheleh Ghahary, Marzieh Rabiei, Sohrab Nasiri, Juozas Padgurskas, Raimundas Rukuiza

**Affiliations:** 1 Department of Chemistry, Najafabad Branch, Islamic Azad University, Najafabad 8514143131, Iran; rahelehghahary@gmail.com; 2 Department of Mechanical, Energy and Biotechnology Engineering, Vytautas Magnus University, Akademija, LT-53361 Kaunas, Lithuania; sohrab.nasiri2017@gmail.com (S.N.); juozas.padgurskas@vdu.lt (J.P.); raimundas.rukuiza@vdu.lt (R.R.); 3 Faculty of Mechanical Engineering and Design, Kaunas University of Technology, Studentu Street 56, LT-51373 Kaunas, Lithuania

**Keywords:** thermally activated delayed fluorescence (TADF), light-emitting electrochemical cells (LECs), organic light-emitting diodes (OLEDs), reverse intersystem crossing (RISC), mechanochromic

## Abstract

Recent advancements in thermally activated delayed fluorescence (TADF) materials and mechanochromic materials have significantly enhanced the efficiency and versatility of light-emitting electrochemical cells (LECs) and organic light-emitting diodes (OLEDs). TADF materials have enabled efficiency improvements, achieving an internal quantum efficiency (IQE) of nearly 100% by utilizing both singlet and triplet excitons. Meanwhile, mechanochromic materials exhibit reversible optical changes upon mechanical stimuli, making them promising for stress sensing, encryption, and flexible electronics. The synergistic integration of TADF and mechanochromic materials in OLEDs and LECs has led to enhanced efficiency, stability, and multifunctionality in next-generation lighting and display technologies. This narrative review explores recent breakthroughs in devices that incorporate both TADF and mechanochromic materials as emitters. Particular attention is given to the molecular design that enable both TADF and mechanochromic properties, as well as optimal device structures and performance parameters. Moreover, this review discusses the only LEC fabricated so far using a TADF-mechanochromic emitter, highlighting its performance and potential. Finally, the report concludes with an outlook on the future commercial applications of these materials, particularly in wearable electronics and smart display technologies.

## 1. Introduction

TADF materials and mechanochromic materials are two classes of materials that have been of immense concern in recent years because of their superior potential for optoelectronic devices. The discovery of TADF materials provided the vision of improving the efficiencies of devices, particularly OLEDs, based on the attainment of near-unity IQE via singlet and triplet exciton harvesting [1,2]. In addition to that, mechanochromic materials also attract special interest because they have the capability to reversibly change their optical features under mechanical stimuli, a quality that is disclosing a range of potential applications ranging from stress sensing to flexible electronics [3,4].

Though both TADF and mechanochromic materials have been successful in their own right, the use of these together and in devices like OLEDs and LECs is a yet-to-be-explored field [5]. Furthermore, recent advancements in photonic materials have revealed the potential of machine learning (ML) and multiphotonic effects to enhance the design and performance of TADF and mechanochromic systems. By enabling efficient analysis of complex datasets and uncovering structure–property relationships, ML offers new strategies for optimizing emission behavior and guiding material discovery [6,7,8]. This review attempts to bridge this gap by suggesting the use of TADF and mechanochromic materials together in these optoelectronic devices. By way of analysis of the molecular design rules and device architectures giving rise to both properties, the review illuminates how the materials might function synergistically to maximize the performance, stability, and multifunctionality of OLEDs and LECs.

**Figure 1:**
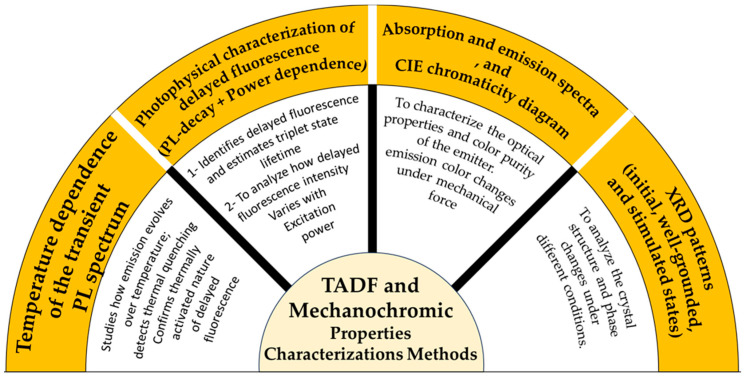
An overview of mentioned TADF and mechanochromic properties characterization methods.

**Figure 2:**
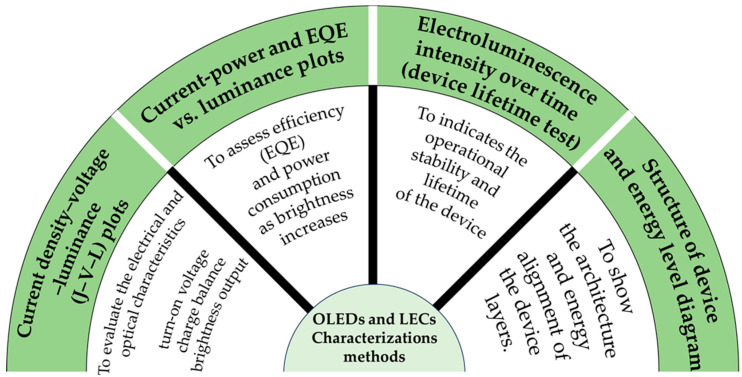
An overview of mentioned devices (OLEDs and LECs) characterization methods.

The paper is presented in a manner where it initially introduces the basic principles and mechanisms of OLEDs and LECs and describes their principal differences among device types. It then describes TADF and mechanochromism mechanisms, and then discusses how integration of these materials into OLED and LEC devices can improve functionalities [9]. The review also features excellent work and developments, including the first LEC ever made to date based on a TADF-mechanochromic emitter, which demonstrates the potential of this promising field [10]. Furthermore, it concludes with an outlook on future commercial applications, particularly in smart display technology and wearable electronics, where these advanced materials will play a critical role (Figure 1 and Figure 2 summarize the TADF/mechanochromic and device characterization methods described later in the manuscript).

## 2. Brief Description of LECs and OLEDs

**Figure 3:**
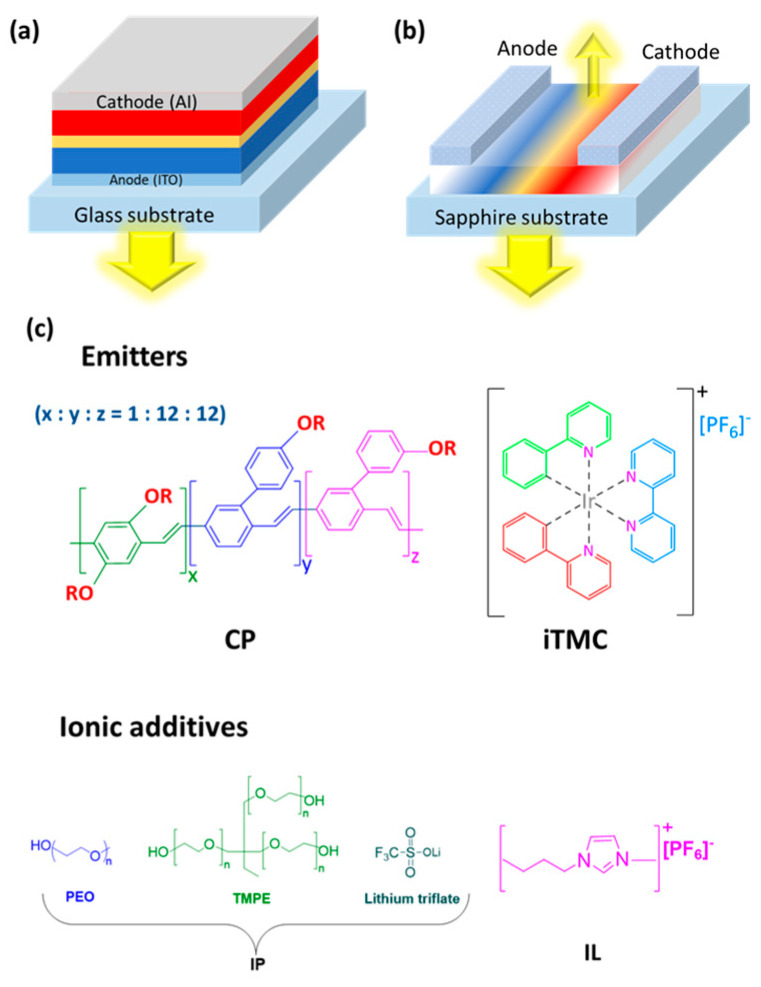
Schematic representations of LEC configurations and materials: (**a**) sandwich configuration, (**b**) surface configuration with interdigitated electrodes, and (**c**) chemical structures of various emitters and ionic additives adapted and redrawn from [19,23].

LECs, an emerging thin-film emissive technology for solid-state lighting, were first developed by Pei et al. in 1996 [11]. Their initial design utilized a luminescent conjugated polymer combined with an ion-conducting polymer, poly(ethylene oxide) (PEO), and lithium triflate as the electrolyte [11]. Taking Figure 3 into account, the glass substrate structure emits light from below and the sapphire substrate structure emits light from above. One has a planar sandwich arrangement, the other is arranged vertically. The lower structure uses an indium tin oxide (ITO) anode at the base, while the upper one has electrodes on the surface. A glass-based device is commonly used for transparent emission, while a sapphire-based device is suitable for directional or microstructured emission [12].

Structurally, LECs are planar, layered devices consisting of an electroluminescent organic semiconductor (OSC) and mobile ions as the active layer, positioned between two metal electrodes. As shown in Figure 3, these electrodes can be arranged in a conventional sandwich structure or with interdigitated configurations [11,13]. The active layer comprises an emitter, such as a conjugated polymer (CP) [11], ionic transition–metal complex (iTMC) [9,11] small molecule (SM) [14], quantum dot (QD) [15], or nanoparticle (NP) [16], blended with ionic additives like ion polyelectrolytes (IP) [17] or ionic liquids (IL) [18] (Figure 3c). The emitter must demonstrate reversible electrochemical behavior and high photoluminescence quantum yield (ϕ) in the solid state, with ions playing a vital role in the operational mechanism [19]. This thin active layer, approximately 100 nm, relies on a substrate for mechanical support [20]. LECs function by creating an electrochemically induced p-i-n junction within the active layer. When a voltage exceeding the OSC’s energy gap is applied across the electrodes, the mobile ions in the active material undergo dynamic and reversible redistribution, enabling doping at the electrode interfaces. This redistribution promotes efficient charge injection, transport, and recombination, resulting in effective light emission. As a result, the current in an LEC consists of both ionic and electronic components [11]. After an initial turn-on phase, light is emitted from the active material and exits through the transparent electrode [20,21,22].

Recently, a new type of unique material for optoelectronic LEC/light-emitting diode (LED) devices appeared: solution-processed hybrid organic–inorganic perovskites (HOIPs) with the chemical formula ABX_3_, where “A” is usually a methylammonium (MA^+^), formamidinium (FA^+^) or cesium ion (Cs^+^), “B” is a lead (Pb^2+^) or tin (Sn^2+^) ion, and “X” is a halogen ion (iodine (I^−^), bromine (Br^−^), or chlorine (Cl^−^)), have emerged as the next generation of light-emitting materials [24,25,26,27,28]. HOIPs are mixed conductors that exhibit both ionic and electronic conductivity. For example, mobile intrinsic point defects (MA^+^, FA^+^, I^−^, and Br^−^ ions) resulting from ionic motion play an important role in the operation of solar cells (SCs) and perovskite-based electrical double layers (EDLs) [29]. The migration of these ions significantly affects the performance of HOIP devices, resulting in high capacitance at low frequencies, electrode polarization, sampling rate-dependent hysteresis, and electroluminescence (EL) color changes during operation. Ion migration in perovskites is primarily due to the low activation energy of anion and cation diffusion in perovskites [30] and extrinsic defects caused by non-stoichiometric and polycrystalline soft lattices with a high density of grain boundaries, although ion redistribution is unfavorable in some conventional devices, ion migration is clearly advantageous for LECs [31,32]. As mentioned above, LECs utilize ion migration as the main working principle, a movement that leads to the formation of a p-i-n structure within the emissive layer (EML) at the interfaces between the EML and the electrodes. The interplay between electronic and ionic currents provides a new way to utilize emitting materials LECs [33,34,35,36,37].

**Figure 4:**
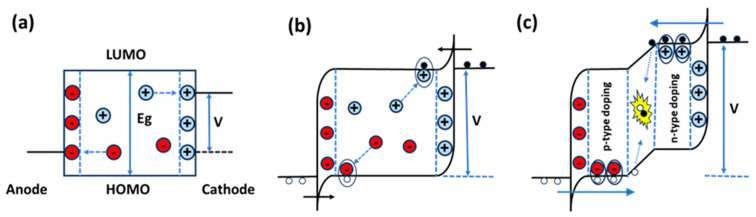
Energy-level diagrams illustrating operation of LECs: (**a**) transient response at eV < E_g_, (**b**) transient response at eV = E_g_, and (**c**) steady-state operation at eV > E_g_. Larger circles depict ions, small open circles represent holes, and small solid circles represent electrons, Adapted and redrawn from [20].

The operation of LECs can be categorized into two regimes depending on the applied voltage. When the voltage is below the energy-gap potential of the OSC (eV < E_g_, typically V < 2.5 V), it is insufficient to trigger redox doping in the OSC. Instead, the mobile ions respond by migrating toward the electrode interfaces, forming thin layers of uncompensated charge known as electric double layers (EDLs) (Figure 4a) [38,39]. The formation of EDLs generates an exceptionally high electric field, approximately 10^9^ V/m [20]. This intense field bends the energy levels at the electrode/active-material interfaces, facilitating the injection of electrons from the cathode into the lowest unoccupied molecular orbital (LUMO, or conduction band) and holes from the anode into the highest occupied molecular orbital (HOMO, or valence band) (Figure 4b).

As the applied voltage reaches the energy gap (E_g_) of the OSC, charge injection commences, marked by the movement of electrons and holes from the electrodes into the active layer. The mobile ions within the active material neutralize these injected charges, stabilizing the charge distribution and facilitating the formation of doped regions (Figure 4b). When the applied voltage exceeds the energy gap (eV > E_g_, typically V > 2.5 V), a p–i–n junction forms (Figure 4c) [38]. In this process, electrons paired with cations are referred to as n-type dopants, while holes paired with anions are termed p-type dopants. This mechanism introduces a high density of charge carriers into the OSC, dramatically increasing its conductivity. Over time, the doped regions expand and eventually merge to form a p–n junction. The remaining potential, not used for charge injection, is dropped across the low-conductive intrinsic region between the doped zones, where light emission occurs [39]. This stage, known as the “turn-on time” of the LEC, marks the point when injected electrons and holes are transported through the doped regions to the junction, where they recombine to form excitons (bound electron–hole pairs). These excitons decay radiatively, emitting photons [20]. The turn-on process depends on the physical displacement of ions within the active layer under the applied voltage, resulting in turn-on times ranging from milliseconds to hours, depending on the ionic conductivity of the active material [39].

To reduce the turn-on time of LECs, it is crucial to minimize the distance that mobile ions must travel within the emissive layer and to maintain a stable, homogeneous mixture of the OSC and the electrolyte. When ions travel shorter distances, the electrochemical doping regions (p-type and n-type) form more quickly near the electrodes, leading to faster charge injection and light emission. At the same time, preventing phase separation between the OSC and the electrolyte ensures uniform ion distribution and efficient recombination, avoiding delayed response or non-uniform emission. Together, these factors enable faster activation and improved performance of LECs [40,41,42,43]. Strategies include using surfactants or selecting electrolytes compatible with the OSC. However, while increasing the concentration of mobile ions accelerates turn-on kinetics, it can compromise device stability and efficiency due to electrochemical degradation [20]. The EQE of LECs is governed by four factors: electrical efficiency (the recombination efficiency of injected charges), radiative efficiency (the fraction of excitons decaying radiatively), the intrinsic photoluminescence (PL) efficiency of the material (linked to the photoluminescence quantum yield (PLQY), and light outcoupling efficiency. Once injection barriers are overcome, nearly all injected charges recombine, but photon trapping within the device limits the fraction of light that escapes to about 30% [23]. Therefore, the emitter’s radiative efficiency and PLQY are critical for achieving high EQE. Upon electron–hole recombination, excitons are formed as either singlets or triplets, with a statistical ratio of 25% singlets and 75% triplets. In most organic materials, triplet excitons are inefficient emitters at room temperature, which restricts the efficiency of fluorescent LECs. Phosphorescent emitters, however, can utilize both singlet and triplet excitons, enabling higher efficiency [39]. More recently, emitters based on TADF have shown promise. By harvesting both singlet and triplet excitons for light emission, TADF-based LECs achieve improved EQE and reduced energy consumption [23].

**Figure 5:**
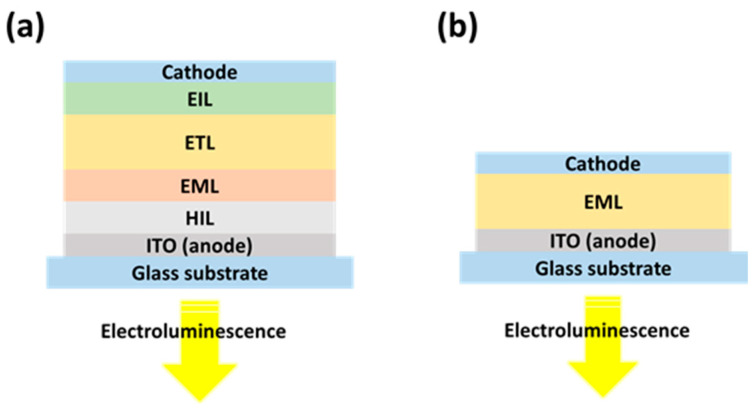
Structure of (**a**) an OLED and (**b**) a LEC, adapted and redrawn from [23].

Unlike LECs, OLEDs do not include mobile ions and use charge injection and recombination within the organic semiconductors alone. OLEDs are organic semiconductor film electroluminescent devices that emit light when a voltage is applied across them [44,45,46]. The structural layers are as follows: transparent anode (typically ITO), hole injection layer (HIL), hole transport layer (HTL), EML, electron transport layer (ETL), electron injection layer (EIL), and metal cathode (such as Al) (Figure 5a) [23,47,48]. Under an application of forward bias, electrons are injected from the cathode to the LUMO of the ETL through the EIL, and holes are injected from the anode to the HOMO of the HTL through the HIL. The charge carriers meet and recombine in the EML to create excitons electron–hole pairs coupled [49,50,51]. Singlet (25%) and triplet (75%) spin states are occupied by excitons in their formation. Only singlet excitons can effectively radiatively decay and give out light in fluorescent OLEDs. Improved IQE is realized through the utilization of phosphorescent emitters incorporating heavy metals (e.g., Ir or Pt complexes) to entrap both triplet and singlet excitons [52,53,54]. TADF emitters have been well received in recent years as a triplet harvesting process via reverse intersystem crossing (RISC) without heavy metal doping [55]. Photon emission due to exciton recombination in the EML is emitted out of the device via the transparent top anode [56]. Unfortunately, due to waveguiding and internal reflection, no more than ~20–30% of the photons can escape, which limits the EQE. Optical outcoupling methods are usually employed to address this loss [57,58]. OLED performance is governed by charge injection efficiency (aided by HIL/EIL), energy level alignment, charge carrier mobility, exciton formation and management, and light outcoupling. These parameters directly impact device efficiency, brightness, and operational stability [59,60].

The thickness of the emissive layer and the particle size of the active materials significantly affect light emission in both OLEDs and LECs. In OLEDs, thin and optimized layers improve exciton recombination, while excessive thickness increases internal resistance and operating voltage [61]. In LECs, thicker layers reduce ionic mobility and delay junction formation. Smaller particles typically enhance film uniformity and charge transport, leading to better emission performance [62,63]. Furthermore, in these LEC devices, the emitting layer serves as the area where charge carriers, electrons, and holes combine to generate light. If the layer is too thick, it can hinder charge transport, increase the distance the ions have to travel (in LECs), and lead to recombination losses or delayed light emission. Conversely, a layer that is too thin cannot support efficient charge injection or uniform light generation, resulting in lower brightness or instability of the device. Similarly, the particle size of the active materials affects the film morphology, surface roughness and interfacial contact with the charge transport layers. Smaller particles generally result in smoother and more uniform films that improve charge mobility and emission uniformity. Larger or aggregated particles, on the other hand, can introduce scattering sites, increase surface defects and cause phase separation, negatively impacting device efficiency and stability. Therefore, optimization of both thickness and particle size is crucial to achieve high-performance electroluminescence devices with fast turn-on time, high brightness, and stable operation [41].

### 2.1. Differences Between LECs and OLEDs

LECs offer several advantages over OLEDs due to their distinct operating principles. LECs are thin, lightweight, and cost-effective devices, with their emission color determined by the energy gap (E_g_) of the OSC [64]. Since the energy gap is dictated by the chemical structure of the OSC, which can be extensively tailored, LECs can be engineered to emit virtually any desired color. LECs are generally more economical than OLEDs due to their simpler structure, lower material costs, and compatibility with solution-based fabrication methods [65,66]. Unlike OLEDs, which require multiple functional layers and high-vacuum deposition techniques, LECs use a single active layer that combines the emitter and ionic species, allowing them to be fabricated through low-cost processes such as spin coating or inkjet printing. Additionally, LECs can operate with air-stable electrodes, eliminating the need for complex encapsulation systems, which further reduces manufacturing costs. While OLEDs offer higher efficiency and longer operational lifetimes, especially in commercial display applications, LECs provide a cost-effective alternative for flexible, printable, and large-area lighting technologies. A key distinction between LECs and OLEDs lies in their charge transport mechanisms. While OLEDs rely solely on the movement of electronic charges, LECs incorporate mobile ions within the light-emitting layer. These ions enable in situ electrochemical doping, facilitating the formation of a p–n junction structure during device operation. Once this p–n junction forms, it creates a low-resistance pathway for charge carriers, leading to sharp turn-on characteristics for both current and light emission [23]. LECs can be operated with switch-on voltages of typically less than 3 V. This is due to an in situ electrochemical doping mechanism in which mobile ions migrate within the emission layer under an applied electric field and form p-doped and n-doped regions near the anode and cathode, respectively. This process creates ohmic contacts at both electrodes, which significantly reduce the barriers to charge injection and enable efficient charge carrier injection and radiative recombination at low voltages [67,68,69]. This mechanism allows LECs to operate with turn-on voltages typically below 3 V, as the electrochemical doping process reduces the turn-on voltage (V_on_) to a level near the energy gap of the active material. Another fundamental difference is LECs’ ability to operate under reverse voltage polarity. The redistribution of mobile ions in the active layer enables the n-doped and p-doped regions to switch positions when the applied voltage is reversed. Additionally, the presence of mobile ions reduces the sensitivity of LECs to active layer thickness, uniformity, and electrode material properties. This flexibility allows the same electrode materials to be used across a range of active layers with varying energy gaps and supports the use of air-stable materials for both the active layer and electrodes. LECs also benefit from a simplified fabrication process. They can be entirely manufactured using solution-processing techniques, requiring only one or two layers of active material to be coated. This makes LECs highly suitable for low-cost, large-scale production [70]. OLEDs typically require vacuum deposition to build multiple layers of different organic materials on a transparent electrode, such as ITO (Figure 5). Furthermore, OLEDs are highly sensitive to the precise thickness and chemical properties of each layer and the electrodes, which adds significant complexity to their fabrication process [11,20,71].

LEC compounds can be dissolved or dispersed in liquids, either in their natural form or after chemical modification with solubilizing groups. These solutions can be processed as standard “inks” and applied using techniques such as spin-coating, scalable printing, or slot-die coating. This versatility allows for efficient, large-scale, solution-based fabrication of flexible LECs through low-cost printing and roll-to-roll manufacturing processes. As a result, LECs offer a significantly lower cost-per-lumen metric compared to OLEDs and LEDs [39,72]. However, despite these advantages, LECs have notable limitations. A primary drawback is their slow response time, which stems from the time required for mobile ions to diffuse and establish the p–n junction. This ion redistribution introduces a delay between the device’s activation and the onset of light emission. Additionally, LECs have a relatively short operational lifetime, which remains a significant challenge for most commercial applications [23,73] (Table 1 summarizes the advantages and disadvantages of OLEDs and LECs).

### 2.2. TADF Mechanism

**Figure 6:**
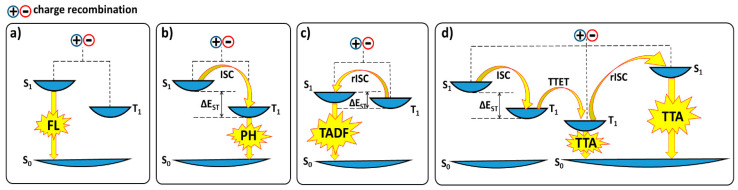
Radiative deactivation pathways existing in (**a**) fluorescent, (**b**) phosphorescent, (**c**) TADF, and (**d**) TTA [76].

The injected charges in light-emitting devices exhibit random spin orientations, leading to the formation of excitons in a 3:1 ratio of triplet (symmetric) to singlet (antisymmetric) states, as governed by spin statistics. In fluorescent (FL) materials with weak spin–orbit coupling, only singlet excitons efficiently undergo a radiative transition to the ground state, limiting light emission to just 25% of the generated excitons (Figure 6a). In contrast, phosphorescent (PH) materials, which typically contain metal complexes, benefit from strong spin–orbit coupling. According to Figure 6b, this coupling facilitates efficient electron transfer from the singlet excited state (S_1_) to the lower-energy triplet state (T_1_) via ISC. ISC occurs when an excited singlet or triplet state transitions to its opposite without radiation, causing the electron’s spin to reverse. This process results in radiative decay from the triplet state to the singlet ground state, releasing a photon of light, a phenomenon known as phosphorescence. Phosphorescence enables both singlet and triplet excitons to contribute to light emission, significantly enhancing overall luminescence efficiency. By utilizing both singlet and triplet excitons, phosphorescent materials can achieve a theoretical IQE of 100% [74,75]. Consequently, exciton statistics dictate the maximum IQE for different materials: up to 25% for fluorescent devices, where only singlet excitons contribute to emission, and up to 100% for phosphorescent devices, which harness both singlet and triplet excitons. While phosphorescence-based OLEDs exhibit high efficiency and long operational lifetimes for red and green emissions, their color purity tends to be lower compared to fluorescent emitters. Additionally, the ISC process, driven by the heavy-atom effect, requires the inclusion of rare and expensive metals like iridium (Ir) or platinum (Pt) [76,77,78]. The theoretical limitation of 25% light emission from excitons in traditional fluorescent devices has been overcome with the development of delayed fluorescence (DF) materials.

DF can generally be classified into two types: E-type, where TADF effectively converts triplet excitons to singlet states, and P-type, where triplet fusion via triplet–triplet annihilation (TTA) converts non-emissive triplet states into emissive singlet states. TADF materials are specifically designed to utilize both singlet and triplet excitons by thermally upconverting non-emissive triplet states (T_1_) to emissive singlet states (S_1_) through a process known as RISC (Figure 6c). This process enables TADF materials to achieve nearly 100% IQE [23,76]. TTA involves the interaction of two triplet excitons. During TTA, one molecule transfers its excited state energy to another, resulting in the first molecule returning to its ground state while the second molecule is promoted to a higher excited state (singlet, triplet, or quintet) (Figure 6d). A perfect TTA process can convert all triplet excitons to singlet states, leading to a theoretical maximum IQE of 62.5% for TTA [76].

**Figure 7:**
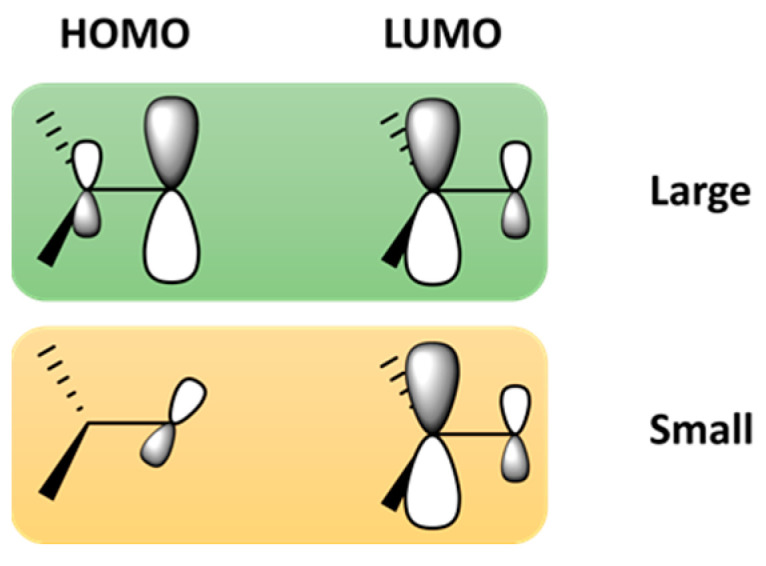
Strategy of realizing small ΔE_ST_ in organic molecules, adapted and redrawn from [77].

TADF, introduced by Adachi et al. using small organic molecules [50], is a method for harvesting triplet excitons and converting them to singlet states by controlling the energy gap (ΔE_ST_) between the singlet and triplet excited states [79]. When this gap is small enough, the triplet state (T_1_) can be converted into the singlet state (S_1_) using only thermal energy from the surroundings through RISC. As depicted in Figure 7, this small ΔE_ST_ can be achieved through precise molecular design, incorporating intramolecular charge transfer between spatially separated donor and acceptor units that are nearly orthogonal to each other. In designing TADF materials, the importance of spin–orbit vibronic coupling must also be considered, in addition to a small ΔE_ST_. While a small ΔE_ST_ is necessary for efficient RISC, it alone is insufficient to guarantee efficient TADF [80]. The process requires strong spin–orbit coupling, which facilitates the transition between the singlet and triplet states. Therefore, effective RISC in TADF materials involves a combination of small ΔE_ST_ and substantial spin–orbit coupling, while maintaining the separation of the HOMO and LUMO wavefunctions to minimize ΔE_ST_ [77,81,82]. For TADF-based systems, smaller particle size and optimized film thickness enhance exciton diffusion and reduce non-radiative losses, which are critical for efficient RISC and delayed fluorescence. Excessive thickness may hinder charge balance and increase triplet quenching [49].

**Figure 8:**
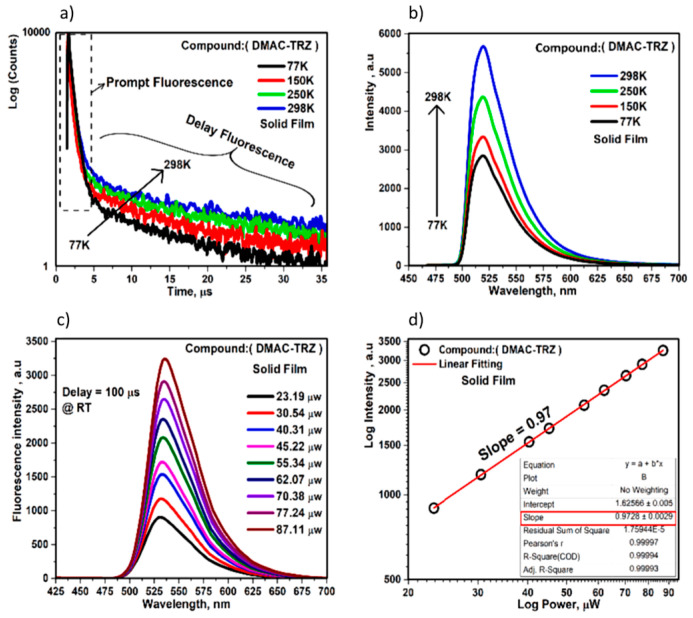
(**a**) Temperature dependence of transient PL-decay spectra, (**b**) temperature dependence of transient PL spectrum, (**c**) power dependence of delayed fluorescence, and (**d**) straight linear fit of delayed fluorescence intensity as a function of excitation power of spin-coated (DMAC-TRZ)-doped compound on solid film [76].

Since TADF emission originates from a triplet state, any process that impacts the population of triplet excitons will also influence the intensity and lifetime of the TADF emission. One such process is the quenching of triplet states by triplet oxygen from the air. Additionally, oxygen acts as a strong triplet quencher, deactivating TADF by depleting triplet populations, while high reorganization energy and suboptimal donor–acceptor molecular design further reduce RISC efficiency. These combined effects serve as key limitations to achieving high-performance TADF emitters [83]. Oxygen interacts with triplet states, reducing their population and thereby quenching the TADF emission. As a result, in both liquid and solid samples (particularly in air-permeable materials), TADF emission is observable only in oxygen-free conditions. In contrast, in air-equilibrated conditions, delayed fluorescence either does not occur or its intensity and lifetime are significantly diminished. Therefore, reliable characterization of TADF materials should include experiments conducted under oxygen-free conditions to obtain accurate results [84,85,86]. The fluorescence decay of a TADF emitter in a degassed solution or a solid film under vacuum typically consists of two components: prompt fluorescence and delayed fluorescence. Prompt fluorescence, which originates from a singlet state, typically has a lifetime in the range of 1–100 ns, similar to conventional fluorescence. Delayed fluorescence, on the other hand, arises from the lowest triplet state (T_1_) through a triplet–singlet transition and has a lifetime ranging from 1 μs to 100 ms. Taking into account Figure 8a, the prompt fluorescence lifetime and intensity change only slightly with temperature, showing longer lifetimes and higher intensities at lower temperatures due to the suppression of non-radiative decay pathways. In contrast, delayed fluorescence is strongly temperature-dependent, with both intensity and lifetime varying significantly with temperature [84,87,88].

To qualitatively confirm if a material exhibits TADF, its photoluminescence lifetime (*τ*) can be studied as the temperature is lowered, typically from 300 K to 77 K (Figure 8a). In TADF materials, the lifetime increases at lower temperatures because RISC is not thermally accessible, and only the T_1_ state is actively participating in the emission process [89,90]. At higher temperatures, the S_1_ state becomes populated according to the Boltzmann distribution, which results in a reduction of the observed lifetime (*τ*). The emission at room temperature typically reflects both the TADF process and a contribution from the T_1_ → S_1_ transition [91,92]. Additionally, another key indicator for evaluating TADF behavior is the bathochromic shift of the emission maxima as the temperature is lowered. This shift occurs because the emission originates from the lower-lying triplet state at lower temperatures. In Figure 8b, the intensity of the PL spectrum decreased with decreasing temperature at 77 K, confirming the TADF mechanism.

TADF is a unimolecular process, meaning that it involves only a single excited molecule in a triplet state. However, in molecules with long triplet lifetimes and relatively large energy gaps (ΔE_ST_), TADF may not be observed, but TTA can occur instead. TTA requires direct contact between two molecules in their triplet excited states. When two triplet excitons interact, they can combine to form a singlet excited state, which subsequently emits delayed fluorescence. Unlike TADF, which is a unimolecular process, TTA is a bimolecular process and is limited by the diffusion rate of the triplet excitons. As such, TTA is also thermally activated. The intensity of TTA is quadratically dependent on the excitation dose, while the intensity of TADF follows a linear dependence (Figure 8d). This difference in power dependence makes power dependence experiments an effective and reliable method for distinguishing between TADF and TTA (Figure 8c). In TADF, being a unimolecular process, the intensity scales linearly with the excitation dose, while in TTA, the intensity scales quadratically. In some cases, TTA and TADF can compete with each other, leading to an intermediate power law dependence, with an exponent between 1 and 2, which reflects the contribution of both processes [84]. While TADF utilizes RISC to convert triplet excitons back to the singlet state for delayed fluorescence, TTA involves the interaction of two triplet excitons to generate a higher energy exciton, which can lead to fluorescence or non-radiative loss. When both mechanisms are active, the emission intensity as a function of excitation power often follows a nonlinear relationship expressed as a power law (I ∝ Pⁿ). For pure TADF, the exponent n is close to 1, reflecting a linear dependence on the excitation density. For pure TTA, n approaches 2 due to its bimolecular nature (two triplets are required). When both TADF and TTA occur simultaneously, the system exhibits an intermediate exponent (1 < n < 2), indicating a mixed contribution from both monomolecular (TADF) and bimolecular (TTA) processes [93].

### 2.3. Mechanochromic Mechanism

**Figure 9:**
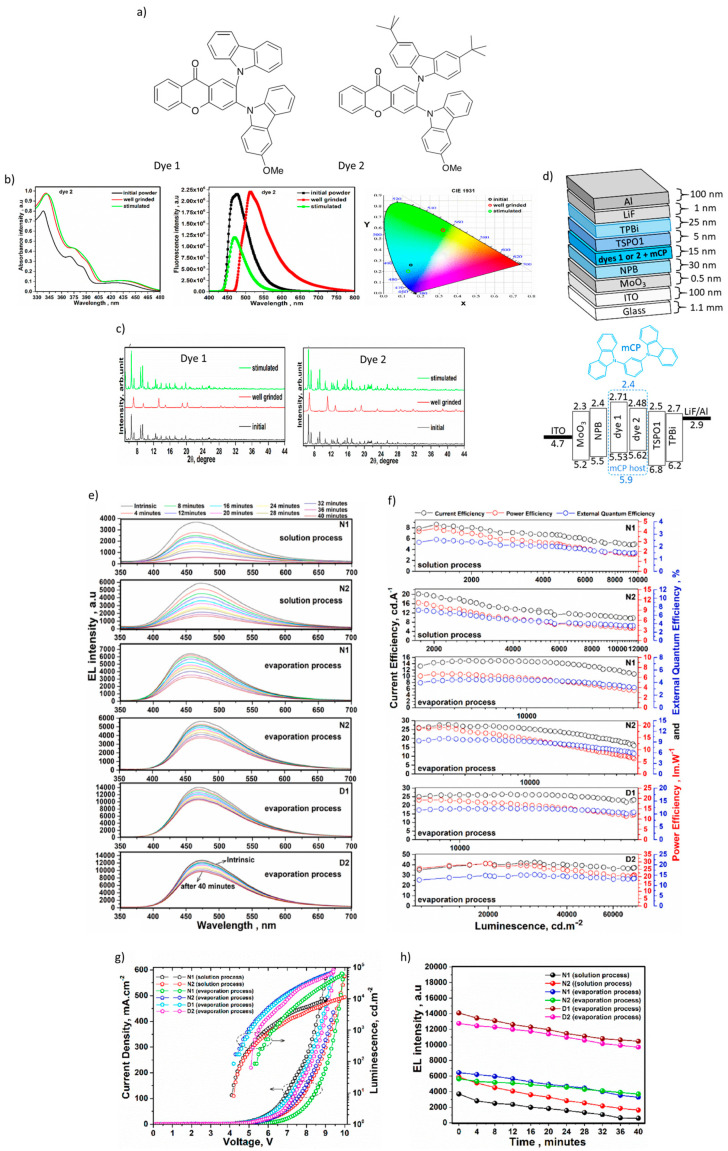
(**a**) Molecular structures of dye 1 and dye 2. (**b**) Absorption and emission spectra, and CIE chromaticity diagram of dye 2 under the well grinded and stimulated modes. (**c**) XRD patterns of dye 1, and dye 2 at initial, well grinded, and stimulated modes. (**d**) Structure of OLEDs and energy level diagram of employing doped (evaporation process). (**e**) Stability of electroluminescence spectra. (**f**) Current–power and external quantum efficiency versus luminance plots of non-doped and doped OLEDs. (**g**) Current density voltage–luminance plots of non-doped and doped OLEDs. (**h**) Variations of electroluminescence intensity over utilization time [96].

**Figure 10:**
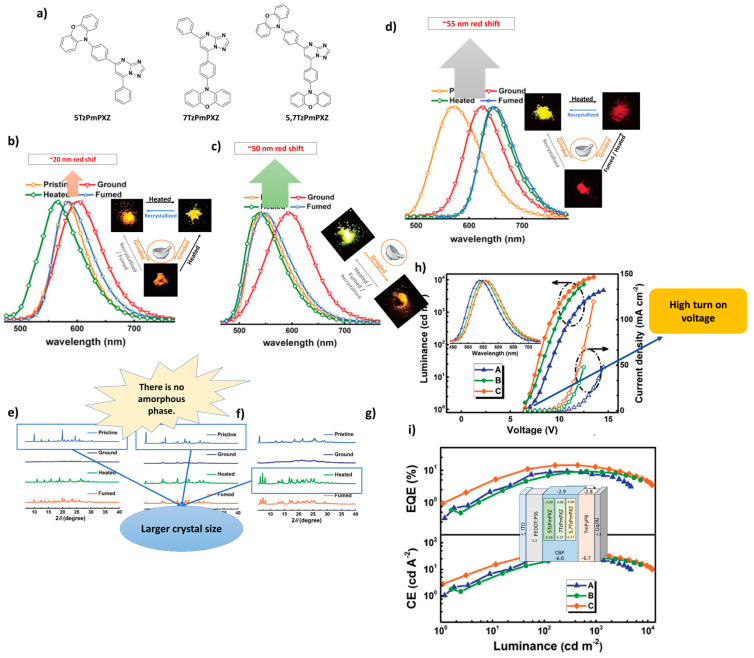
(**a**) Molecular structures of target compounds, Photographs and PL spectra of (**b**) 5TzPmPXZ, (**c**) 7TzPmPXZ, and (**d**) 5,7TzPmPXZ in response to external stimuli. Photographs were taken under UV irradiation with 365 nm. (grinding with a mortar and a pestle; heating at 150 °C for 5TzPmPXZ and 7TzPmPXZ, 200 °C for 5,7TzPmPXZ; fuming with CH_2_Cl_2_ vapor; recrystallization from n-hexane/CHCl_3_), PXRD patterns of (**e**) 5TzPmPXZ, (**f**) 7TzPmPXZ, and (**g**) 5,7TzPmPXZ, (**h**) Luminance–voltage–current density curves for devices A, B, and C (inset: the normalized EL spectra of devices). (**i**) External quantum efficiency and current efficiency versus luminance curves for devices A, B, and C (inset: The energy level diagrams for the devices A, B, and C). Reanalyzed and modified from data reported in [102].

Mechanochromism refers to the reversible changes in the visible optical properties of materials, such as color or intensity in response to mechanical stimuli like pressure, stretching, grinding, or rubbing. Mechanochromic compounds are capable of altering their luminescence color when subjected to mechanical forces, making them particularly valuable in applications such as strain sensing, structural health monitoring, and secure encryption technologies [94,95,96]. The color change in these materials typically arises from two primary mechanisms: (1) molecular interactions that result in aggregation or disaggregation of the molecules under mechanical force, and (2) alterations in the conjugation length of molecules when subjected to stress [97]. The mechanical force can trigger chemical reactions, leading to the formation or breaking of covalent bonds, or it can induce structural changes in the molecules, such as conformational shifts. On a larger scale, these molecular interactions influence intermolecular forces, causing aggregation, which in turn alters the emission properties of the material. In addition, light interference effects are observed in mechanochromic photonic crystals, which consist of regularly spaced particles within a soft, deformable matrix. In mechanochromic systems, particle size and film morphology directly influence aggregation behavior, which governs the color and intensity of emission. Thinner, well-dispersed films help maintain reversible luminescence, while thicker or highly aggregated domains may lead to irreversible emission changes or reduced sensitivity [98,99]. Smaller particle sizes and uniform film morphology favor more controlled molecular packing, while larger or irregular structures can lead to disordered aggregation. The way in which molecules aggregate, e.g., by forming H-aggregates or J-aggregates, directly influences the photophysical properties, with H-aggregates generally causing a blue-shifted and weaker emission and J-aggregates leading to a red-shifted and stronger fluorescence. When mechanical force is applied, this molecular packing is disrupted, often resulting in a transition between crystalline and amorphous phases or a change in aggregate types, which changes the emission color or intensity. As a result, the mechanochromic response is very sensitive to the initial particle size and film morphology, which determine how the molecules interact and respond to external stress [100]. Mechanical deformation of materials changes their optical path length, contributing to their mechanochromic behavior [95]. If a compound exhibits mechanochromic potential, the application of external pressure or tension can enhance its emission properties, with corresponding shifts in the excitation or emission spectra depending on the material’s sensitivity to the applied stimuli. By comparing the photophysical properties of these materials before and after mechanical stimulation, mechanochromic behavior can be identified. For example, dyes synthesized by Nasiri et al. demonstrated a red shift in absorption bands after grinding, attributed to reduced intermolecular distances and rotational adjustments of molecular planes due to mechanical stress. Conversely, increased intermolecular distances can lead to a blue shift in some cases (Figure 9b) [96]. X-ray diffraction (XRD) analysis offers valuable insights into the structural changes induced by mechanical forces. According to Bragg’s equation (nλ = 2dsinϴ, where n is a positive integer, λ is the wavelength of the incident wave, d is the interplanar spacing of the crystal, and ϴ is the angle of incidence), shifts or changes in XRD peaks indicate alterations in the interplanar spacing (d), which are directly influenced by mechanical processes such as grinding. The planar density has a significant influence on mechanochromic behavior, as it determines how densely the molecules in a film or crystal are packed and interact. A high planar density leads to strong intermolecular interactions, such as π-π stacking and exciton coupling, which make the system more sensitive to mechanical stimuli. When force is applied, this dense packing can be disrupted, triggering phase transitions e.g., from crystalline to amorphous state or changes in aggregate state, both of which alter the photophysical properties of the material. These structural changes affect the delocalization of excitons and the energy band structures, resulting in visible shifts in emission color or intensity. Therefore, areal density plays a crucial role in determining the reactivity and emission behavior of mechanochromic materials under external stress [101]. Zeng et al. studied the powder-XRD patterns of three mechanochromic materials before and after grinding (Figure 10e–g) [102]. The pristine sample showed sharp and intense diffraction peaks, but after grinding for several minutes, these peaks disappeared, suggesting that grinding converted the pristine material to an amorphous form. Interestingly, when the ground sample was heated, the diffraction peaks reappeared but with different 2θ values. Furthermore, after fuming the ground sample with CH_2_Cl_2_ vapor, the diffraction peaks partly restored to the original pattern of the pristine material, highlighting the dynamic structural changes induced by mechanical and chemical stimuli [102].

In mechanochromic systems, mechanical stimuli induce conformational changes, particularly affecting the dihedral angle between donor and acceptor units. These changes influence the electronic structure and consequently the emission behavior. However, the exact donor–acceptor angle at which prompt fluorescence or delayed fluorescence (including TADF or general delayed emission) occurs is typically unknown and cannot be directly measured through common structural techniques such as XRD [103].

What we do know is that at certain angles, delayed fluorescence can occur, although not necessarily through a TADF mechanism. For instance, in our observations, some materials exhibited delayed emission with a lifetime of around 999 ns, which is not in the microsecond range typical for TADF, suggesting a non-TADF delayed fluorescence pathway.

At specific donor–acceptor angles (hypothetically, for example, ~32°), both HOMO and LUMO orbitals may become accessible and aligned in a way that facilitates charge transport, hole/electron recombination, and even polarization effects. These processes are likely to be mechanically induced due to the flexible molecular conformation and packing in mechanochromic systems. Because such structural transitions are subtle and dynamic, they are often not easily characterized by conventional methods.

Additionally, the probability of these electronic phenomena increases with the incorporation of multiple donor units, which enhances conformational flexibility and increases the likelihood of favorable orbital alignment under mechanical stress [104]. A comparison of the advantages and disadvantages of TADF and mechanochromic-based materials in OLEDs and LECs is summarized in Table 2.

### 2.4. Integration of TADF and Mechanochromic Materials in OLEDs and LECs

Nasiri et al. [96], developed two novel TADF and mechanochromic dyes based on a D–A–D′ configuration, where a xanthene derivative serves as the acceptor, and two asymmetric carbazole derivatives function as donor units, which molecular structures are shown in Figure 9a. Based on Figure 9b,c, the photoluminescence spectrum of dye 2 under the well grinded mode is red-shifted (43 nm) and the intensity is increased; interpretation of this event is connected to the intermolecular coupling. Moreover, the XRD patterns of the well grinded dyes were significantly shifted. The best-performing OLED (D2), based on dye 2 (with the substitution of tert-butyl derivative) doped with the 50 wt.% of 1,3-Bis (N-carbazolyl) benzene (mCP) as a host material, demonstrated a low turn-on voltage of 5.09 V at 63.68 cd/m^2^, achieving peak values for brightness, current efficiency, power efficiency, and external quantum efficiency at 72,565 cd/m^2^, 37.32 cd/A, 20.99 lm/W, and 13.41%, respectively. The maximum main peaks of emission spectra for dye 2 were observed at 518 nm and 472 nm under the well grinded and stimulated modes, respectively. The highest PLQY values (41%) belonged to the spin-coated doped dye 2 on the solid films. Moreover, the ΔE_ST_ value of deposited dye 2 on the solid state was registered as 0.37 eV. According to Figure 9d, the optimized device composed ITO/MoO_3_ (0.5 nm)/NPB (30 nm)/50 wt% emitters:mCP (15 nm)/TSPO1 (5 nm)/TPBi (25 nm)/LiF (1 nm)/Al, coated by evaporation process. In this structure, molybdenum oxide (MoO_3_) and lithium fluoride (LiF) served as the hole and electron injection layer, respectively. The N,N′-Di(1-naphthyl)-N,N′-diphenyl-(1,1′-biphenyl)-4,4′-diamine (NPB) and Poly(3,4-ethylenedioxythiophene)-poly(styrenesulfonate) (PEDOT:PSS) have played the role of a hole transport and the hole injection layer; while, the PEDOT:PSS had an adhesion character in the solution process. Also, diphenyl [4-(triphenylsilyl) phenyl] phosphine oxide (TSPO1) and 1,3,5-tris (Nphenylbenzimidazol-2-yl) benzene (TPBi) were utilized as the hole blocking and electron transporting layer, respectively. From Figure 9e–h, we can see the characterization of all OLEDs fabricated in this research, doped and non-doped, with using dye 1 and dye 2 via evaporation and solution process [96].

In 2018 Zeng et al. developed three multifunctional mechanochromic luminescent (MCL) TADF materials—5TzPmPXZ, 7TzPmPXZ, and 5,7TzPmPXZ—by incorporating phenoxazine with the electron-acceptor core [1,2,4]triazolo [1,5-a]pyrimidine (TzPm) (Figure 10a) [102]. Referring to Figure 10b–g, mechanochromic properties of compounds are shown in photographs were taken under UV irradiation with 365 nm, before and after stimuli. Moreover, shift of the PL spectra of compounds and changing the PXRD pattern after stimuli are another prove of the mechanochromic properties of these materials. The 5,7TzPmPXZ compound exhibited high PLQY of 0.66 in CBP (4,4′-di(9H-carbazol-9-yl)-1,1′-biphenyl) as a host (0.7 wt%) and a small singlet–triplet energy gap (ΔE_ST_) of 0.06 eV. The solution-processed OLED fabricated using 5,7TzPmPXZ demonstrated excellent performance, with a main emission peak at 560 nm. The device, configured as indium tin oxide/poly(3,4-ethylenedioxythiophene)-doped poly(styrenesulfonate) (PEDOT:PSS) (30 nm)/CBP:TADF emitter (0.7 wt%, 40 nm)/1,3,5-tri(m-pyrid-3-yl-phenyl)benzene (TmPyPB) (60 nm)/8-hydroxyquinolinolato-lithium (Liq) (1 nm)/Al (100 nm) (Figure 10i), achieved a maximum current efficiency (CE) of 41.9 cd/A, a maximum luminance (L_max_) of 12,210 cd/m^2^, and a EQE of 14.3%. All results achieved by using three materials are shown in Figure 10h,i [102].

**Figure 11:**
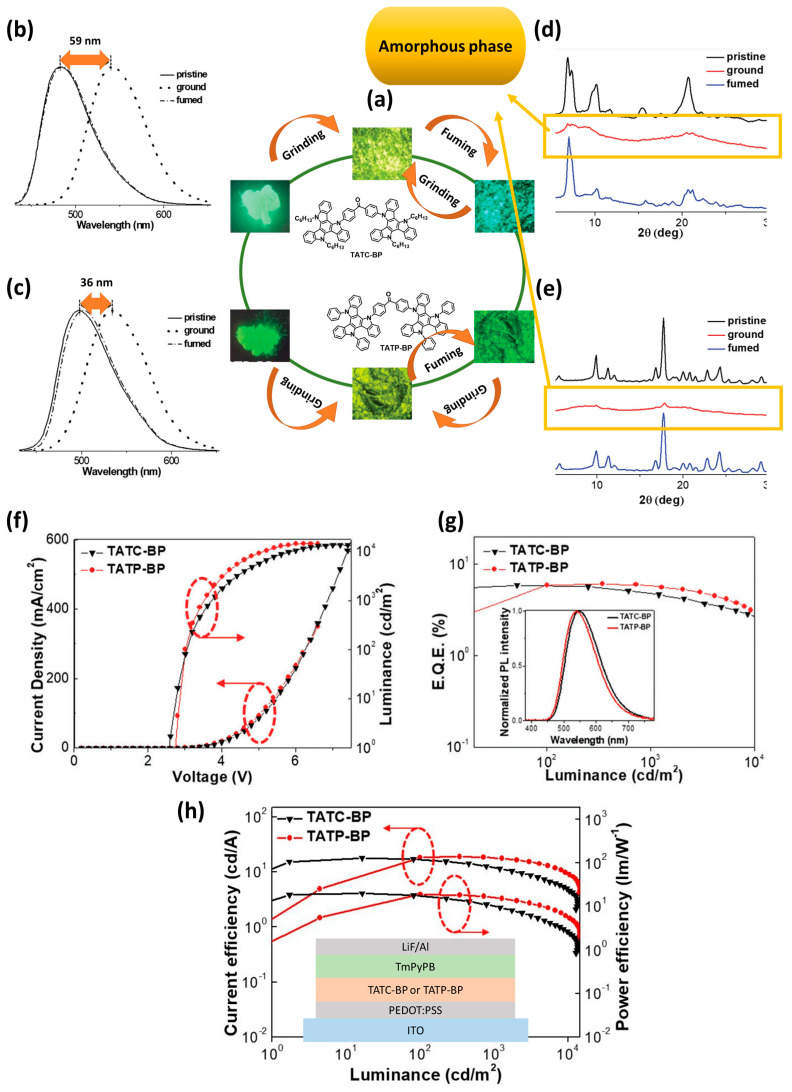
(**a**) Molecular structures of TATC-BP and TATP-BP, and Fluorescent images (under UV illumination) of the pristine crystalline, ground, and vapor fumed powders of TATC-BP and TATP-BP. Normalized PL spectra of TATC-BP (**b**) and TATP-BP (**c**) (excitation wavelength: 365 nm), Powder X-ray diffraction patterns of TATC-BP (**d**) and TATP-BP (**e**) before and after grinding, and after fuming treatment of the ground solid with dichloromethane, (**f**) Current density–voltage–luminance and, (**g**) external quantum efficiency–luminance characteristics. Inset: EL spectra of the OLED devices at 1000 cd m^−2^, (**h**) Current efficiency/luminance/power efficiency curves of the nondoped devices of TATC-BP and TATP-BP, Inset: device configuration. Reanalyzed and modified from data reported in [105].

Chen et al. [105], synthesized two new solution-processable triazatruxene-based derivatives,bis(4-(10,15-dihexyl-10,15-dihydro-5H-diindolo[3,2-a:30,20-c]carbazol-5-yl)phenyl)methanone (TATC-BP) and bis(4-(10,15-diphenyl-10,15-dihydro-5H-diindolo [3,2-a:30,20-c]carbazol-5-yl)phenyl)methanone (TATP-BP), which combine triazatruxene as the electron donor and benzophenone as the electron acceptor (Figure 11a). These derivatives exhibit TADF, aggregation-induced emission (AIE), and MCL property. Taking into consideration Figure 11a, fluorescent images (under UV illumination) of the pristine crystalline, ground, and vapor fumed powders of TATC-BP, and TATP-BP demonstrate the mechanochromic behavior of these derivatives. Furthermore, the shift observed in the PL spectra and PXRD patterns in Figure 11b–e confirms the mechanochromic properties of these material. The optimized OLED device fabricated using TATP-BP, with configuration shown in Figure 11h, with a small ΔE_ST_ of 129 meV, exhibited distinct delay components with excited state lifetimes (τ_d_) of 0.91 µs. In neat films, TATP-BP showed a photoluminescence (PL) maximum at 520 nm, with a PL quantum yield (ϕ_PL_) of 24.2%. The solution-processed non-doped OLED using TATP-BP, configured as ITO/PEDOT (25 nm)/TATP-BP (25 nm)/TmPyPB (55 nm)/LiF (1 nm)/Al (150 nm), demonstrated enhanced electroluminescence (EL) performance, with emission at 541 nm, a V_on_ of 2.7 V, peak power efficiency (PE) of 19.2 lm/W, a maximum current efficiency of 18.9 cd/A, a peak EQE of 6.0%, and an impressively low EQE roll-off of just 3.3% at 1000 cd/m^2^. Additionally, doped OLEDs were fabricated using the same device configuration for both emitters in a H2 host (30 wt.% doping concentration). Devices based on TATC-BP and TATP-BP achieved maximum EQEs of 15.9% and 15.4%, along with maximum current efficiencies of 48.1 cd/A and 46.4 cd/A, respectively. The characterizations of the fabricated OLEDs are presented in Figure 11f–h [105].

**Figure 12:**
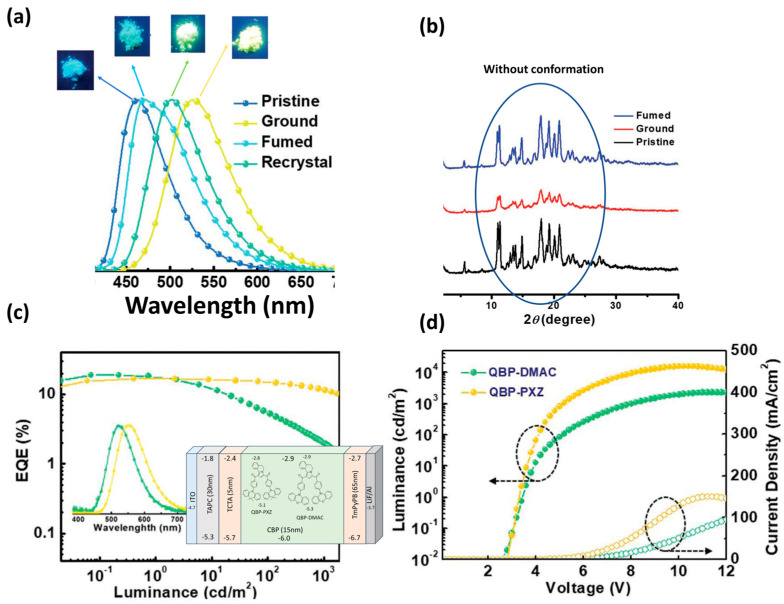
(**a**) Photographs and PL spectra of QBP-DMAC in response to external stimuli. Photographs were taken under UV irradiation at 365 nm. (grinding with a mortar and a pestle; fuming with CH_2_Cl_2_ vapor; recrystallization from n-hexane/CH_2_Cl_2_). (**b**) PXRD patterns of QBP-DMAC, (**c**) EQE versus luminance curves of the devices. Inset: Normalized EL spectra and device structure. (**d**) Luminance–voltage–current density curves of the devices. Reanalyzed and modified from data reported in [106].

Zheng et al. designed and synthesized two butterfly-shaped, multifunctional TADF emissive molecules, QBP-DMAC and QBP-PXZ (Figure 12c), incorporating a novel acceptor unit, (5,6-dihydropyrrolo [2,1-a]isoquinoline-1,3-diyl)bis(phenylmethanone) (QBP), with distinct donor groups: 9,9-dimethyl-9,10-dihydroacridine (DMAC) and 10H-phenoxazine [106]. The photoluminescence (PL) spectrum of QBP-DMAC exhibited a structureless emission peak at 508 nm. For QBP-DMAC, the calculated values include a small ΔE_ST_ of 0.33 eV, an emission lifetime (τ_p_) of 13.0 ns, and a delayed lifetime (τ_d_) of 1.87 ms. The PLQY for QBP-DMAC in CBP films (9 wt%) reached 0.78 under argon conditions. Based on the results of photographs, PL spectra and the XRD pattern of QBP-DMAC before and after stimuli (Figure 12a,b), QBP-DMAC also demonstrated multicolor MCL. An OLED device fabricated with this molecule in an optimized structure (ITO/TAPC (30 nm)/TCTA (5 nm)/CBP (9 wt%, 15 nm)/TmPyPB (65 nm)/LiF (1 nm)/Al (100 nm)), shown in Figure 12c, exhibited enhanced electroluminescent (EL) performance, with emission at 523 nm, a V_on_ of 3.6 V, peak PE of 55.8 lm/W, a maximum current efficiency of 56.8 cd/A, L_max_ of 2264 cd/m^2^, and a peak EQE of 18.8%. Figure 12c,d illustrate the performance of the two OLEDs that were fabricated by QBP-DMAC and QBP-PXZ [106].

**Figure 13:**
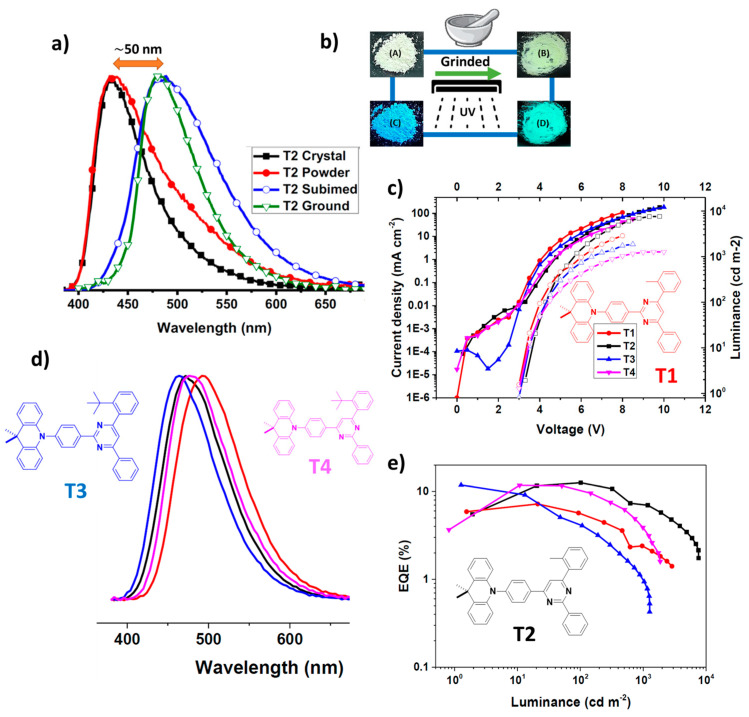
(**a**) Photoluminescence spectra of T2 in single crystal (black), powder (red), sublimation state (blue) and ground state (green) respectively, (**b**) Photo-graphic images of the color and luminescence changes of T2 in response to mechanical grinding: (A) and (C) unground sample, (B) and (D) ground sample. Photographs were taken under ambient light and UV irradiation (365 nm), (**c**) Current density-voltage-luminance, (**d**) EL spectra of the blue OLEDs, (**e**) external quantum efficiency-luminance plots. Reanalyzed and modified from data reported in [107].

Ganesan et al. [107], synthesized four molecules with both TADF and mechanochromic properties, incorporating dimethyl acridine as the electron donor and linking it to diphenyl pyrimidine acceptors in either symmetrical or asymmetrical configurations (Figure 13). The PLQY of the charge transfer emission for T2, the best-performing emitter in the fabricated OLED, was 43% in degassed CH₂Cl₂, compared to 16% in aerated conditions. For T2 in CH₂Cl₂, the key photophysical parameters were as follows: ΔE_ST_ of 77 meV, charge transfer emission maximum (λ_CT_) at 566 nm, intersystem crossing rate constant (k_isc_) of 3.93 × 10⁷ s^−1^, and radiative intersystem crossing rate constant (k_risc_) of 6.42 × 10⁵ s^−1^. According to Figure 13a,b, the maximum peak of PL spectra of T2 is red shifted to 483 nm after applying the mechanical force such as grinding. The best-performing OLED device, with the configuration ITO/TAPC (50 nm)/mCP:10 wt% dopant (15 nm)/DPEPO:10 wt% dopant (15 nm)/TmPyPB (60 nm)/LiF (1 nm)/Al (100 nm), showed electroluminescent (EL) emission at 490 nm, a V_on_ of 3 V (Figure 13c), a L_max_ of 7385 cd/m^2^, and an EQE of 14.2%. These results were achieved using a 12 nm emitting layer with T2 as the emitter. Figure 13c–e illustrate the current density vs. voltage–luminance characteristics, EQE vs. luminance performance, and EL spectra of the blue OLEDs employing T1–T4 as emitters [107].

**Figure 14:**
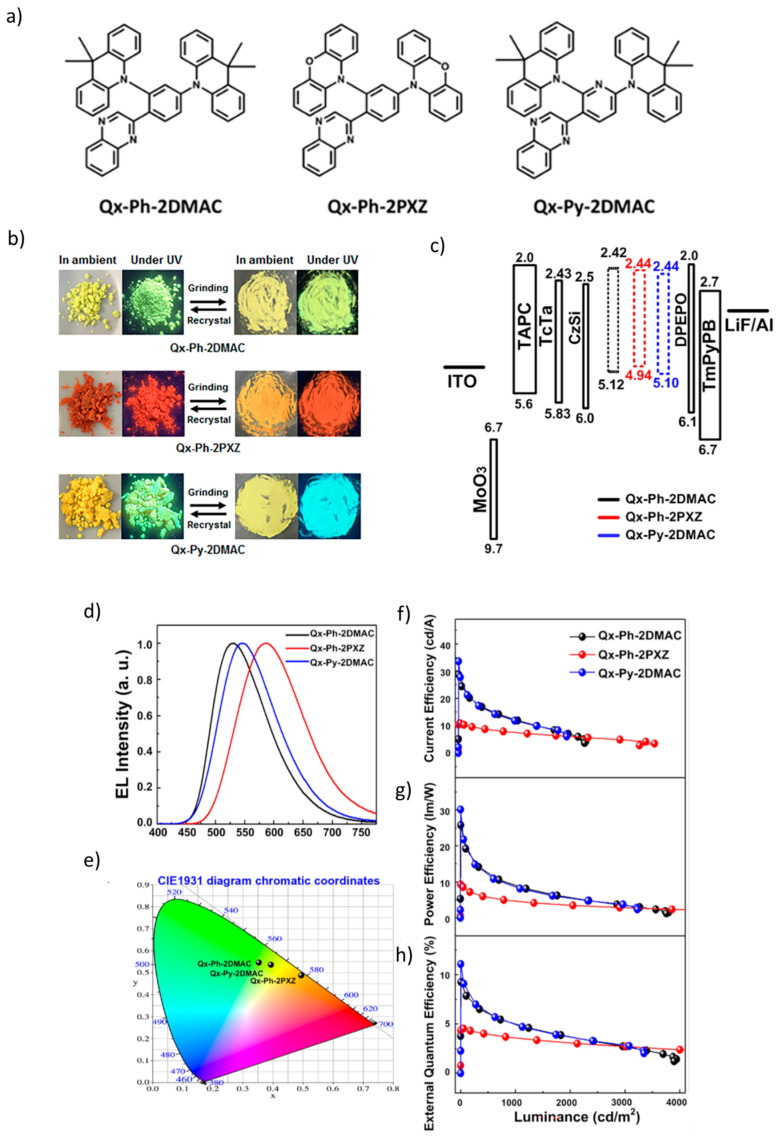
(**a**) Molecular structures of organic emitters. (**b**) Photographs of mechanochromic emission in response to external stimuli. (**c**) Energy band diagram of non-doped OLEDs. (**d**) Normalized electroluminescence spectra. (**e**) CIE 1931 coordinates. (**f**) Current efficiency, (**g**) power efficiency, and (**h**) external quantum efficiency of triplet-harvesting non-doped OLEDs [108].

Kim et al. [108], synthesized three asymmetric HAF D–π–A type multifunctional organic emitters—Qx-Ph-2DMAC, Qx-Ph-2PXZ, and Qx-Py-2DMAC—by introducing variations in linker and donor groups to investigate the intramolecular hydrogen bonding (IHB) effect (Figure 14a). As shown in Figure 14b, Qx-Py-2DMAC exhibited notable multifunctionality, including AIE, MCL, delayed TADF, and room-temperature phosphorescence (RTP) in the solid state. It demonstrated enhanced radiative properties with a high quantum yield of 0.94 in neat film, and emission peaks at 548 nm in dilute toluene and 550 nm in vacuum-deposited film at 300 K, with an experimental ΔE_ST_ of 0.06 eV. According to Figure 14c, a non-doped OLED using Qx-Py-2DMAC, with the structure ITO/MoO₃ (50 Å)/TAPC (450 Å)/TcTa (50 Å)/CzSi (20 Å)/Qx-Py-2DMAC/DPEPO (30 Å)/TmPyPB (500 Å)/LiF (10 Å)/Al (1200 Å), achieved an impressive current efficiency of 33.6 cd/A, a V_on_ of 2.82 V, L_max_ of 3336 cd/m^2^, power efficiency of 30.1 lm/W, and EQE of 11.1%. In Figure 14d,e, normalized electroluminescence spectra, and CIE 1931 coordinates of the fabricated OLEDs are shown, respectively. Moreover, the current efficiency, power efficiency, and external quantum efficiency versus the luminance of the devices are depicted in Figure 14f–h [108].

**Figure 15:**
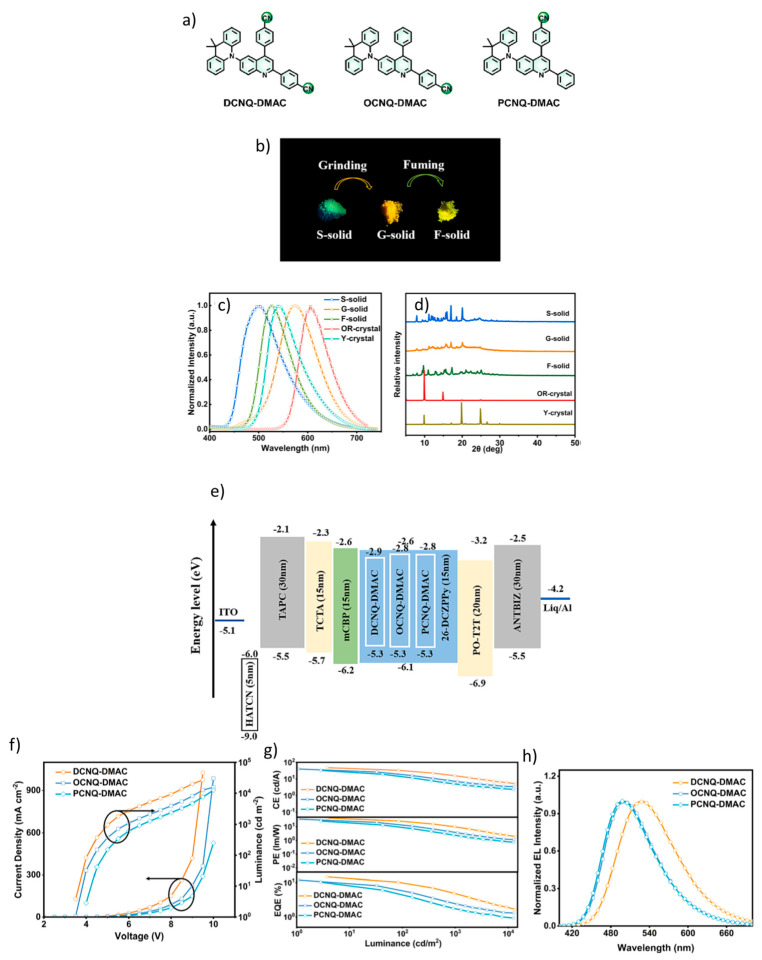
(**a**) Molecular structure of the emitters. (**b**) Photographs of DCNQ-DMAC taken under a 365 nm UV lamp after crushing or exposure to dichloromethane vapors. (**c**) Normalized fluorescence spectra of DCNQ-DMAC in different states of aggregation. (**d**) XRD curves of DCNQ-DMAC in different states of aggregation. (**e**) Doped device structure and energy level diagram of the materials. (**f**) Current density–voltage–luminance curves for the three compounds. (**g**) Current efficiency, power efficiency, and EQE as a function of luminance for the three compounds. (**h**) Normalized electroluminescence (EL) spectrum [109].

Hu et al. developed and synthesized three novel organic multifunctional luminescent emitters (Figure 15a): 4,4′-(6-(9,9-dimethylacridin-10(9H)-yl)quinolin-2,4-diyl)dibenzonitrile (DCNQ-DMAC), 4-(6-(9,9-dimethylacridin-10(9H)-yl)-4-phenylquinolin-2-yl)benzonitrile (OCNQ-DMAC), and 4-(6-(9,9-dimethylacridin-10(9H)-yl)-2-phenylquinolin-4-yl)benzonitrile (PCNQ-DMAC). These emitters exhibit a combination of TADF, AIE, and polymorphism. As shown in Figure 15b–d, DCNQ-DMAC exhibited remarkable three-color MCL behavior with high contrast (Δλ_em,max_ = 76 nm). The small singlet–triplet energy gap (ΔE_ST_) was calculated to be 0.02 eV for DCNQ-DMAC, 0.07 eV for OCNQ-DMAC and 0.12 eV for PCNQ-DMAC. All emitters showed strong absorption peaks around 320 nm, with featureless yellow-green and greenish emissions observed at 546 nm for DCNQ-DMAC, 516 nm for OCNQ-DMAC, and 524 nm for PCNQ-DMAC. As shown in Figure 15f,g, the highest performing OLED was achieved by doping DCNQ-DMAC into a 2,6-DCzPPy host with a doping concentration of 12 wt%. This device delivered impressive performance metrics: EQE: 14.6%, maximum luminescence (L_max_): 13,576 cd/m^2^, Maximum CE_max_: 47.7 cd/A, Maximum PE_max_: 42.9 lm/W. All devices showed green emission with peak values at 528 nm, 497 nm and 500 nm for DCNQ-DMAC, OCNQ-DMAC, and PCNQ-DMAC, respectively (Figure 15h). The architecture of the OLED device consisted of the following layers (Figure 15e): ITO/HAT-CN (5 nm)/TAPC (30 nm)/TCTA (15 nm)/mCBP (10 nm)/2,6-DCzPPy: emitter (15 nm)/POT2T (20 nm)/ANT-BIZ (30 nm)/Liq (2 nm)/Al. These results underline the extraordinary potential of DCNQ-DMAC and its derivatives for high-performance OLED applications [109].

**Figure 16:**
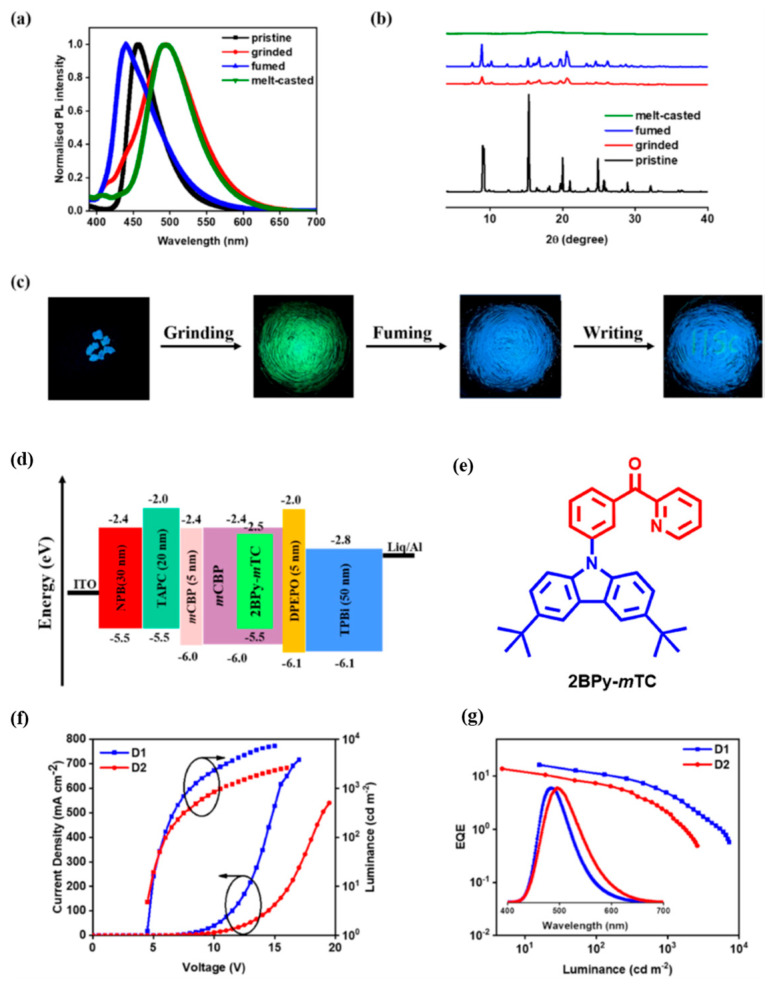
(**a**) Steady-state (PL) photoluminescence spectra of 2BPy-mTC. (**b**) Powder X-ray diffraction pattern (PXRD) of 2BPy-mTC. (**c**) Photographs showing MCL color changes observed under UV irradiation (365 nm) in response to external stimuli. (**d**) Architecture of device and energy level diagram. (**e**) Molecular structure of 2BPy-mTC. (**f**) Current density–voltage–luminance (J-V-L) curves. (**g**) EQE/luminance curves (EQE-L) of devices with normalized EL spectra [110].

Yadav et al. [110], designed and synthesized a multifunctional emitter, 2BPy-mTC (Figure 16e), with benzoylpyridyl (2BPy) as the acceptor and 3,6-di-tert-butyl-9H-carbazole (TC) as the donor. This emitter exhibits TADF, with ΔE_ST_ values of 0.20 eV in a pure film and 0.11 eV in a doped film (5 wt.% 2BPy-mTC:mCBP). The PLQY is 42% in the pure film and 59% in the doped film in vacuum. As shown in Figure 16c, 2BPy-mTC exhibits MCL in response to external stimuli such as grinding, smoking, and melt casting. Initially, it emits blue light with a λ_max_ of 456 nm in its pure form, but after grinding the emission shifts to the green with a λ_max_ of 495 nm. The emission returns to its original state when exposed to dichloromethane vapors (CH₂Cl₂). These grinding-induced changes were confirmed by powder X-ray diffraction (PXRD) measurements (Figure 16b). As shown in Figure 16d, OLEDs were fabricated with doped films of 2BPy-mTC as an EML with the following structure: ITO/N,N′-di(1-naphthyl)-N,N′-diphenyl-(1,1′-biphenyl)-4,4′-diamine (NPB) (30 nm)/4,4′-Cyclohexylidenebis[N,N-bis(4-methylphenyl)benzenamine] (TAPC) (20 nm)/5 wt.% 2BPy-mTC:mCBP (10 nm, D1)/Bis [2-(diphenylphosphino)phenyl]ether oxide (DPEPO) (5 nm)/2,2′,2′’-(1,3,5-benzinetriyl)-tris(1-phenyl-1H-benzimidazole) (TPBi) (50 nm)/Lithium 8-hydroxyquinolinolate (Liq) (2 nm)/Aluminum (Al) (100 nm). The doped device (D1) achieved a maximum EQE_max_ of 16.3%, with an emission peak at 484 nm. The device achieved a L_max_ of 7266 cd/m^2^, a maximum CE_max_ of 39.4 cd/A and a maximum PE_max_ of 24.8 lm/W (Figure 16f,g) [110].

**Figure 17:**
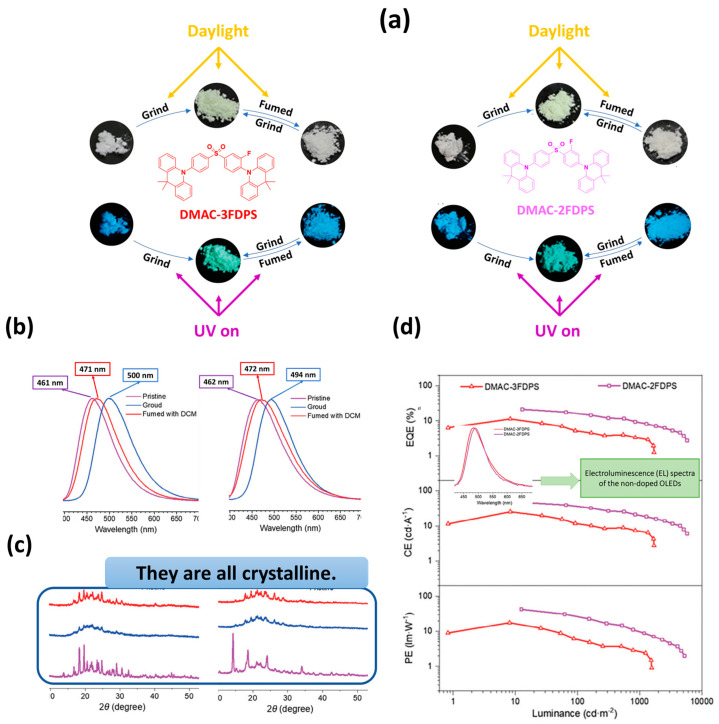
(**a**) Molecular structures of DMAC-3FDPS and DMAC-2FDPS, and photographs of the two isomers in response to external stimuli, taken in daylight and UV irradiation (365 nm). (**b**) Photoluminescence spectra (PL) of the two isomers in response to external stimuli, taken in daylight and UV irradiation (365 nm). (**c**) XRD patterns of the two isomers in different states, (**d**) EQE, CE and PE luminance curves, Inset: Electroluminescence (EL) spectra of the non-doped OLEDs. Reanalyzed and modified from data reported in [111].

In 2024, Ge et al. introduced two new multifunctional isomers, DMAC-2FDPS and DMAC-3FDPS, which exhibit a wide range of remarkable properties, including AIE, MCL, RTP, and TADF. Figure 17a shows the molecular structures of these two unsymmetrical fluorinated diphenyl sulfone acridine derivatives derived from the classical efficient TADF molecule DMAC-DPS. The ΔE_ST_ values for DMAC-2FDPS and DMAC-3FDPS determined by phosphorescence spectra were 0.03 eV and 0.04 eV, respectively, indicating highly efficient RISC processes. DMAC-2FDPS and DMAC-3FDPS show featureless emissions with peaks at 469 nm and 479 nm, respectively, in solution and strong PL intensities with emission peaks at 485 nm and 475 nm in pure films. Figure 17a,b, as well as PXRD measurements (Figure 17c), show the MCL features of both isomers in different states of aggregation. The highest performing non-doped OLED was fabricated using DMAC-2FDPS and exhibited an exceptionally high PLQY of up to 93% in a pristine film. The architecture of the device was as follows: ITO/PEDOT:PSS (30 nm)/mbis(N-carbazolyl)benzene (mCP) (20 nm)/EML (30 nm)/1,3,5-Tris(N-phenylbenzimidazol-2-yl)benzene (TPBI) (40 nm)/LiF (1 nm)/Al (100 nm). As shown in Figure 17d, the electroluminescence (EL) spectrum for the non-doped DMAC-2FDPS-based device peaked at 488 nm. The device exhibited impressive EL performance metrics, including a maximum CE_max_ of 46.8 cd/A, a maximum efficiency (PE_max_) of 42.0 lm/W and a maximum EQE_max_ of 21.2% (Figure 17d) [111].

**Figure 18:**
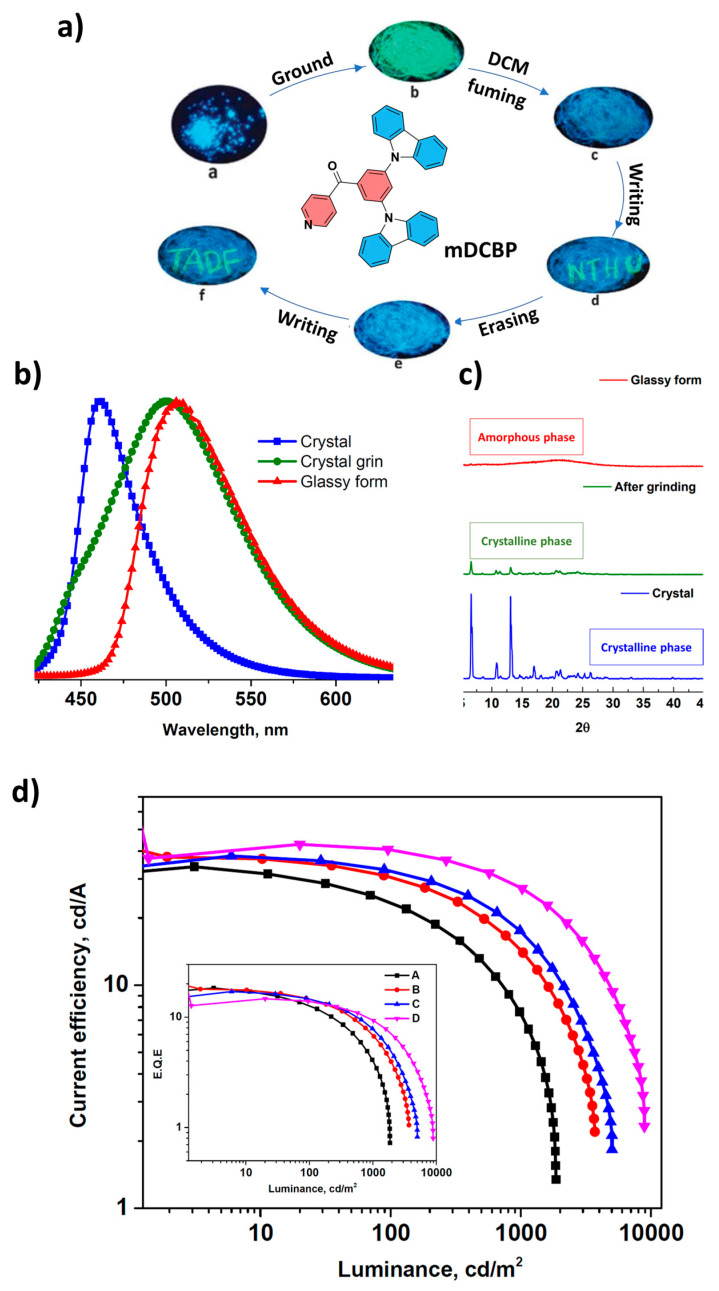
(**a**) Molecular structure of mDCBP, and photos of mDCBP in its different states: (a) crystalline, (b) after grinding, (c) after solvent diffusion, (d) writing of “NTHU”, (e) erasure of letters by solvent diffusion and (f) writing of “TADF” on the material. (**b**) Emission spectra of mDCBP in crystalline, amorphous and glassy form (insets: corresponding photos under UV light). (**c**) Powder X-ray diffraction (PXRD) patterns of mDCBP. (**d**) Current efficiency versus luminance of devices A–D (inset: EQE of devices A–D). Reanalyzed and modified from data reported in [112].

Rajamalli et al. [112], developed a TADF emitter, (3,5-di(9H-carbazol-9-yl)phenyl)(pyridin-4-yl)methanone (mDCBP) (Figure 18a), which has a benzoylpyridine core as electron accepting unit and two meta-carbazolyl groups as electron donating units. The emitter exhibited a very small singlet–triplet energy gap (ΔE_ST_) of 0.06 eV and a PLQY of 90% in a co-doped film (DPEPO:mDCBP (10%)). As shown in Figure 18b, the color of the luminescence of mDCBP changes from blue (λ_max_ = 460 nm) in the crystalline phase to green (λ_max_ = 510 nm) in the amorphous, glassy form when exposed to external stimuli. Furthermore, the powder X-ray diffractogram (PXRD) of mDCBP (Figure 18c) shows intense, sharp peaks for the crystalline form, with peak intensities significantly reduced after grinding. Figure 18a shows that this molecule exhibits reversible, switchable emission when alternating between mechanical force and solvent treatment. To investigate the electroluminescence properties of mDCBP, multilayer OLEDs were fabricated with mDCBP-doped films as the EML. Devices A–D were fabricated with different mDCBP concentrations using the following device structure: ITO/NPB (40 nm)/mCP (10 nm)/DPEPO: mDCBP (X wt%) (30 nm)/PPT (5 nm)/TmPyPb (60 nm)/LiF (1 nm)/Al (100 nm), where x = 10, 15, 20, 30, and the corresponding devices were labeled device A, B, C and D, respectively. Device A (blue emission) and device D (green emission) showed impressive performances. Device A achieved: EQE: 18.4%, Maximum brightness (L_max_): 8900 cd/m^2^, Switch-on voltage: 3.6 V, CE: 34.0 cd/A, PE: 26.5 lm/W. The results underline the potential of mDCBP as a TADF emitter for high-power OLEDs, with blue emission from device A and green emission from device D (Figure 18d) [112].

**Figure 19:**
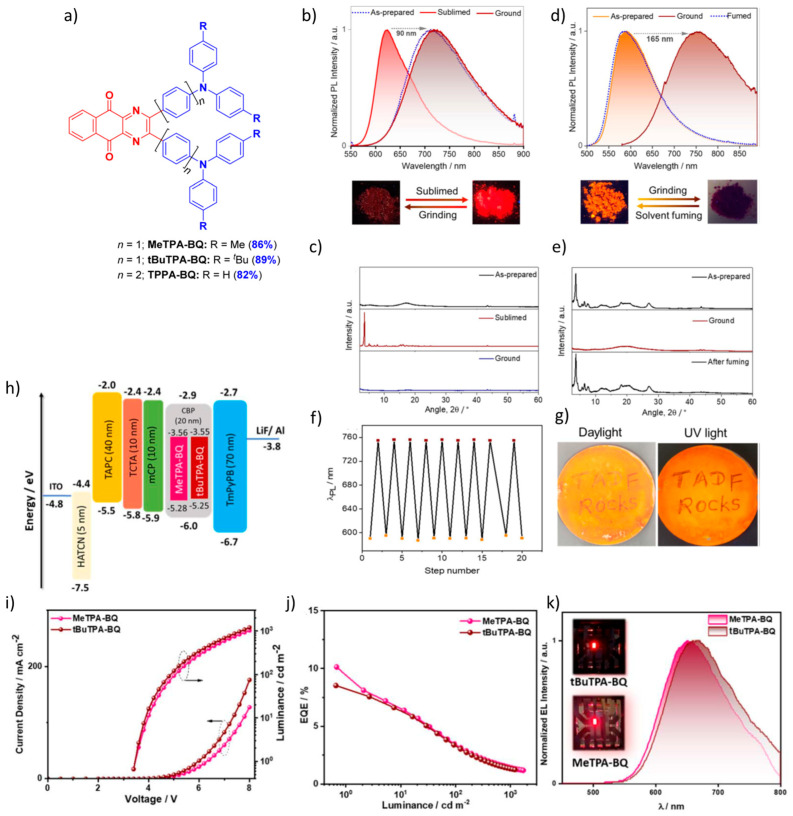
(**a**) Molecular structure of target emitters. (**b**) Photoluminescence (PL) spectra (λ_exc_ = 450 nm) of prepared, sublimated, and polished samples of tBuTPA-BQ, as well as corresponding photos under UV light (λ_exc_ = 365 nm). (**c**) PXRD patterns of prepared, sublimed, and ground samples of tBuTPA-BQ. (**d**) PL spectra (λ_exc_ = 450 nm) of prepared, ground, and EtOAc-coated samples of TPPA-BQ, as well as corresponding photos under UV light (λ_exc_ = 365 nm). (**e**) PXRD pattern of TPPA-BQ samples in initial state, milled, and smoked in EtOAc. (**f**) Repeated change of photoluminescence emission wavelength with mechanical pressure and EtOAc treatment. (**g**) Demonstration of writing and erasing on filter paper, with photographs taken under daylight and UV light (λ_exc_ = 365 nm). (**h**) Energy level diagram of materials used in devices. (**i**) Current density and luminance as a function of voltage for devices. (**j**) EQE curves as a function of luminance for devices. (**k**) Electroluminescence spectra (EL) of devices (inset: photos of the EL of devices) [113].

Sudhakar et al. developed three multifunctional compounds—MeTPA-BQ, tBuTPA-BQ and TPPA-BQ (Figure 19a)—based on a hybrid acceptor, benzo[g]quinoxaline-5,10-dione, designed for emission by TADF. These compounds emit in the red region and exhibit TADF with photoluminescence quantum yields (Φ_PL_) of 42% at 650 nm (MeTPA-BQ), 41% at 670 nm (tBuTPA-BQ) and 39% at 625 nm (TPPA-BQ) in 2 wt% doped films in 4,4′-bis(N-carbazolyl)-1,1′-biphenyl (CBP), with ΔE_ST_ values of 0.01 eV, 0.01 eV and 0.10 eV, respectively. As shown in Figure 19b,d, tBuTPA-BQ and TPPA-BQ exhibit significant PL shifts of about 98 nm and 165 nm, respectively, after milling the crystalline samples, while a smaller PL shift of 10 nm is observed for MeTPA-BQ. Interestingly, the photophysical properties of tBuTPA-BQ do not return to their original state when exposed to solvent vapors (hexane, Et₂O, DCM, THF, EtOAc) or when the ground powder is heated to 200 °C. In contrast, TPPA-BQ regains its original photophysics upon fuming with EtOAc, as confirmed by PXRD analysis (Figure 19c,e). Remarkably, the grinding and smoking cycles for TPPA-BQ were repeated 20 times, showing a high reproducibility of the phase transformations without significant fatigue (Figure 19f). MeTPA-BQ and tBuTPA-BQ were used as emitters in vacuum-deposited bottom-emitting OLEDs using an optimized device structure: ITO/HATCN (5 nm)/TAPC (40 nm)/TCTA (10 nm)/mCP (10 nm)/emitting layer (20 nm)/TmPyPB (70 nm)/LiF (0.7 nm)/Al (100 nm). The devices achieved the following performance figures: MeTPA-BQ (650 nm): Maximum EQE_max_: 10.1%, CE_max_: 5.88 cd/A, PE_max_: 5.43 lm/W. tBuTPA-BQ (670 nm): Maximum EQE_max_: 8.5%, CE_max_: 4.69 cd/A, PE_max_: 4.33 lm/W. These results, which are shown in Figure 19h–k, underline the potential of these compounds as TADF-based emitters for red OLED applications [113].

**Figure 20:**
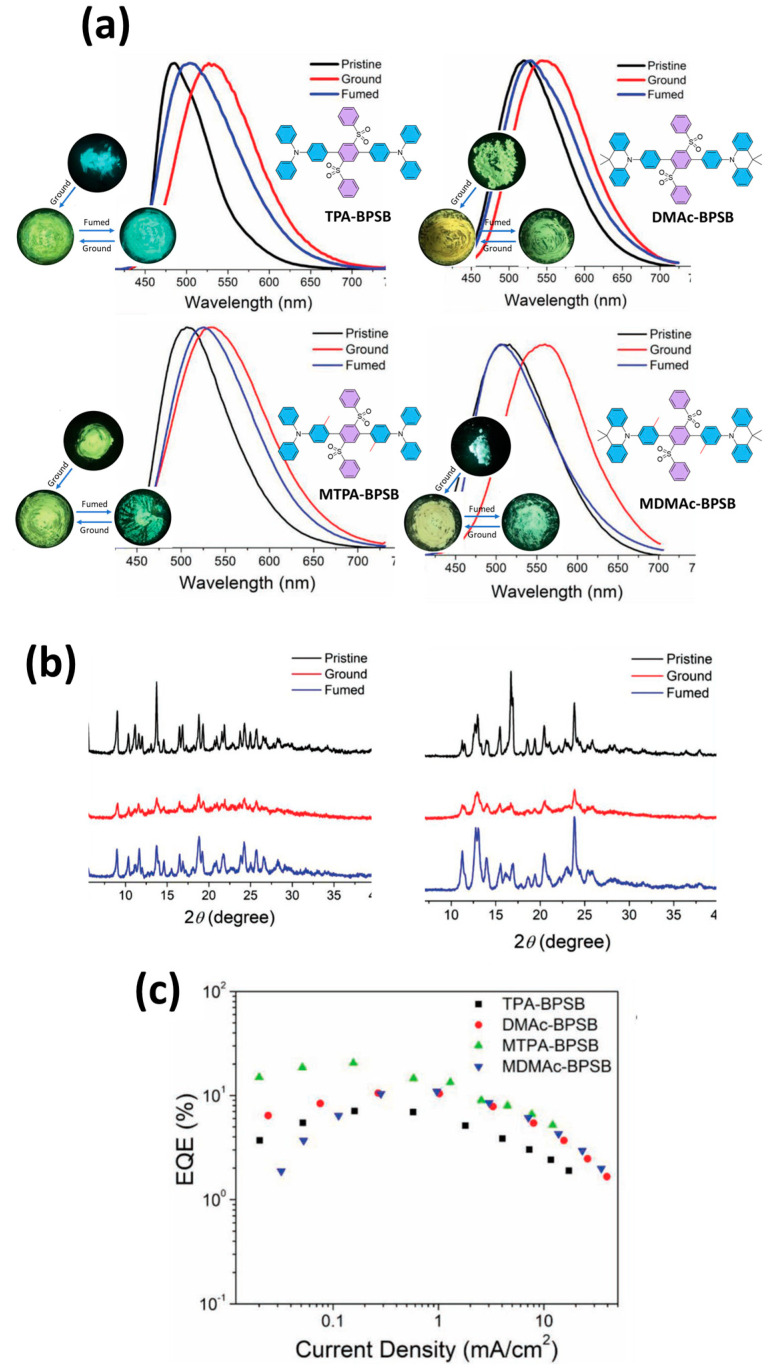
(**a**) Molecular structures of the compounds, and PL spectra and photographs of TPA-BPSB, DMAc-BPSB, MTPA-BPSB and MDMAc-BPSB under different external stimuli, and the photographs were taken under UV ir-radiation at λexc = 365 nm. (**b**) PXRD patterns of MTPA-BPSB and MDMAc-BPSB at room temperature under different external stimuli. (**c**) EQE current density curves of the devices. Reanalyzed and modified from data reported in [114].

Zhou et al. synthesized four cross-shaped molecules: TPA-BPSB, DMAc-BPSB, MTPA-BPSB, and MDMAc-BPSB (Figure 20a), all of which have a common acceptor, bis(phenylsulfonyl)benzene (BPSB), and different donor segments. TPA and MTPA are diphenylamine derivatives, while DMAc and MDMAc are 9,9-dimethylacridine derivatives. These molecules showed distinct TADF properties with green emission both in solution (λ_max_ = 516–546 nm) and in the solid state (λ_max_ = 480–520 nm, in a polymethyl methacrylate (PMMA) film doped with 5 wt%). The ΔE_ST_ values were estimated as: TPA-BPSB: 0.18 eV, DMAc-BPSB: ≈0.02 eV, MTPA-BPSB: 0.07 eV, MDMAc-BPSB: 0.01 eV. The PLQY values of the doped layers were: TPA-BPSB: 87%, DMAc-BPSB: 47%, MTPA-BPSB: 76%, MDMAc-BPSB: 43%. As shown in Figure 20a, the red shift of the emission peaks after grinding illustrates the mechanochromic luminescence properties of these TADF molecules. The PXRD patterns (Figure 20b) confirm the inherent mechanochromic properties of the compounds. Using CzAcSF (10-(4-((4-(9H-carbazol-9-yl)phenyl)-sulfonyl)phenyl)-9,9-dimethyl-9,10-dihydroacridine) as a host, solution-processed OLEDs with the following configuration were prepared: ITO/PEDOT:PSS (40 nm)/CzAcSF:dyes (7 wt%, 50 nm) (TPA-BPSB: device I; DMAc-BPSB: device II; MTPA-BPSB: device III; MDMAc-BPSB: device IV)/DPEPO (9 nm)/TmPyPB (40 nm)/CsF (1.2 nm)/Al (120 nm). The fabricated devices exhibited featureless EL spectra with emission peaks at 504–528 nm. Device II (MTPA-BPSB) showed the best performance and achieved: Maximum CE_max_: 61.6 cd/A, Maximum PE_max_: 37.2 lm/W, Maximum EQE_max_: 20.5%. These results (Figure 20c) underline the exceptional performance of OLEDs based on MTPA-BPSB [114].

**Figure 21:**
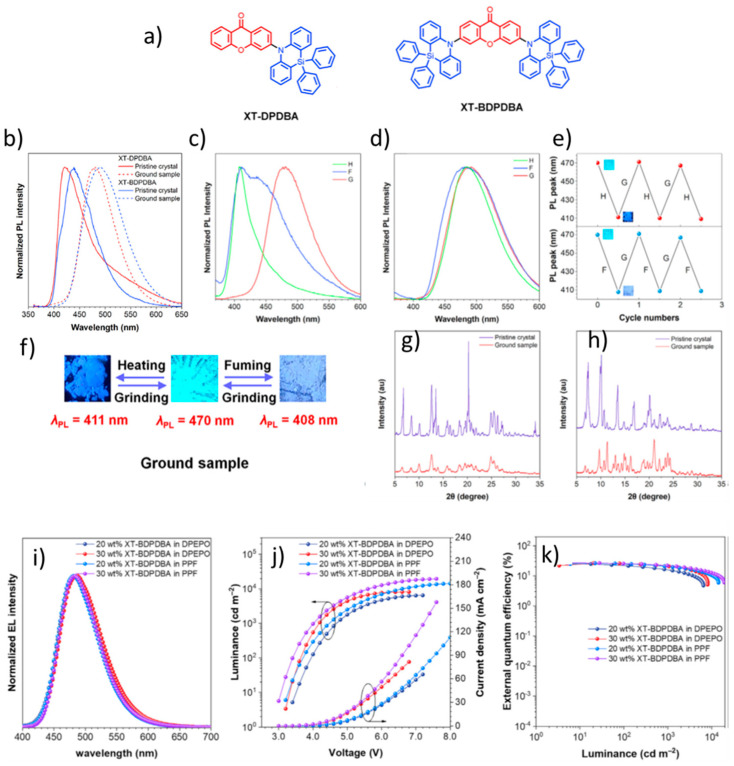
(**a**) Molecular structures of XT-DPDBA and XT-BDPDBA. (**b**) Photoluminescence (PL) spectra of untreated crystals and ground samples. (**c**) PL spectra of ground samples of XT-DPDBA in response to external stimuli (H: heating at 200 °C for 1 h, F: fuming with CH₂Cl₂ for 1 h, G: grinding with a mortar for 30 min). (**d**) PL spectra of milled samples of XT-BDPDBA under same excitation conditions. (**e**) Repeated mechanochromic behavior of XT-DPDBA: grinding and heating (top) and grinding and smoking (bottom), with corresponding photographs of samples under 365 nm UV irradiation. (**f**) Photographs of samples after grinding, heating, and smoking. (**g**) PXRD patterns of crystals and milled samples of XT-DPDBA. (**h**) PXRD patterns of crystals and ground samples of XT-BDPDBA. (**i**) Electroluminescence (EL) spectra of XT-BDPDBA at 10 mA-cm^−2^. (**j**) Luminance–voltage–current density (L–V–J) curves of XT-BDPDBA. (**k**) EQE and luminance curves of XT-BDPDBA [115].

In 2022, He et al. reported two blue emitters, XT-DPDBA and XT-BDPDBA, which consist of the DPDBA donor and XT (xanthenone) acceptor (10-dihydrodibenzo[b,e][1,4]azasiline) (Figure 21a). Doped films of XT-DPDBA and XT-BDPDBA emit weak emissions with peaks at 477 nm and 486 nm, respectively, and exhibit low photoluminescence quantum yields (Φ_PL_) of 97% and 91%. The calculated ΔE_ST_ values are 0.063 eV for XT-DPDBA and 0.025 eV for XT-BDPDBA. Both compounds show aggregation-induced delayed fluorescence and remarkable MCL. Pristine crystals of XT-DPDBA and XT-BDPDBA emit deep blue light with peaks at 421 nm and 439 nm, respectively. During grinding, the emissions shift significantly from deep blue to sky blue (421 to 480 nm for XT-DPDBA and 439 to 492 nm for XT-BDPDBA) (Figure 21b–f). PXRD measurements confirm the MCL behavior of the compounds, as shown in Figure 21g,h. Doped OLEDs based on XT-DPDBA and XT-BDPDBA were prepared with the following configuration: ITO/HATCN (5 nm)/TAPC (50 nm)/TCTA (5 nm)/mCP (5 nm)/EML (20 nm)/DPEPO (5 nm)/TmPyPB (30 nm)/LiF (1 nm)/Al (120 nm). The EML consisted of doped films of the molecules of the 2,8-bis(diphenylphosphoryl)dibenzo[b,d]furan (PPF) host or the DPEPO host with doping concentrations of 20 wt% and 30 wt%. The PPF-doped OLED based on XT-BDPDBA with a doping concentration of 30 wt% exhibited the best performance, including: turn-on voltage: 3.0 V, electroluminescence peak: 484 nm, L_max_: 19,319 cd/m^2^, CE_max_: 55.0 cd/A, PE_max_: 57.5 lm/W, EQE_max_: 27.0% (Figure 21i–k) [115].

**Figure 22:**
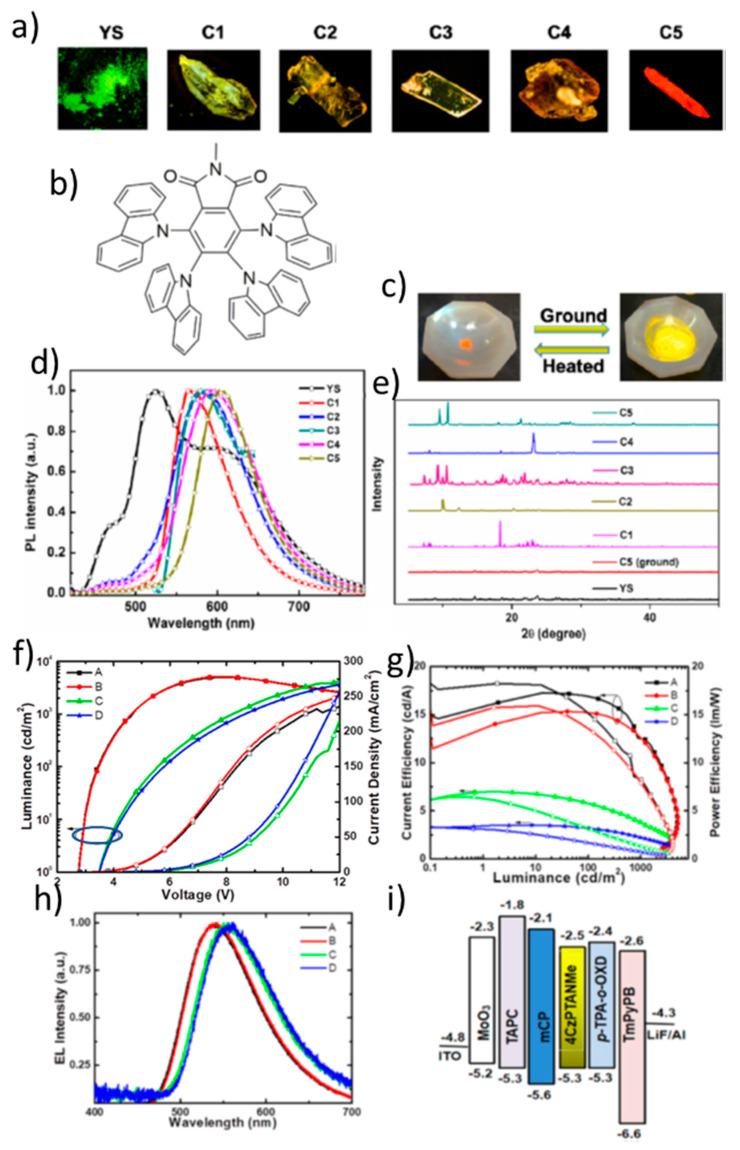
(**a**) Photos of five aggregates of 4CzPTANMe under UV excitation (λexc = 365 nm). (**b**) Molecular structure of 4CzPTANMe. (**c**) Fluorescence image of C5 in a dish (left: as prepared, right: ground with DCM). (**d**) Fluorescence spectra (FL) of 4CzPTANMe in different solid states. (**e**) XRD spectra of 4CzPTANMe in different solid states. (**f**) Luminance–voltage–current density (L–V–J) curves. (**g**) Curves of current efficiency and power efficiency as a function of luminance. (**h**) Normalized electroluminescence (EL) spectra. (**i**) Schematic energy level diagrams of devices [116].

Li et al. reported a novel emitting material, 4,5,6,7-tetrakis-carbazol-9-yl-2-methyl-isoindole-1,3-dione (4CzPTANMe), in which carbazole (Cz) serves as a donor and 2-methyl-isoindole-1,3-dione (PTANMe) as an acceptor (Figure 22b). This emitter shows solvent-induced polymorphism, TADF and MCL. 4CzPTANMe exists in six solvent-induced aggregation states, including five different crystalline forms with emission ranging from green to red. After removal of the solvent, the C5 form shows an efficient TADF with a low ΔE_ST_ of 0.03 eV and a high fluorescence quantum yield (Φ_FL_) of 0.40. As shown in Figure 22a, the yellow solid form (YS) of 4CzPTANMe shows a multicolored emission with a main peak at 526 nm, while the five crystalline forms show a broad, featureless fluorescence with emission peaks at 566 nm (C1), 586 nm (C2), 587 nm (C3), 594 nm (C4), and 602 nm (C5) (Figure 22d). Interestingly, 4CzPTANMe shows remarkable MCL properties. As shown in Figure 22c, C5 is converted to YS by grinding with DCM, while YS is converted back to C5 by thermal treatment (170 °C for 5 s). The PXRD patterns of milled C5 and YS confirm the reversible MCL behavior (Figure 22e). To evaluate the electroluminescence (EL), multilayer OLED devices (A–D) were fabricated with doped films as EML. The structure of the devices was: ITO/MoO₃ (1 nm)/TAPC (40 nm)/EML (20 nm)/TmPyPB (40 nm)/LiF (1 nm)/Al (100 nm) (Figure 22i). In devices A–D, 4CzPTANMe was used in p-TPA-o-OXD or mCP hosts with doping concentrations of 6 or 8 wt%. All devices showed a greenish-yellow emission as shown in Figure 22h. The best performing device, device A, based on 6 wt% 4CzPTANMe doped in p-TPA-o-OXD, achieved: Emission peak: 538 nm, Maximum CE_max_: 15.9 cd/A, Maximum PE_max_: 18.2 lm/W, Maximum EQE_max_: 5.96% (Figure 22f,g) [116].

**Figure 23:**
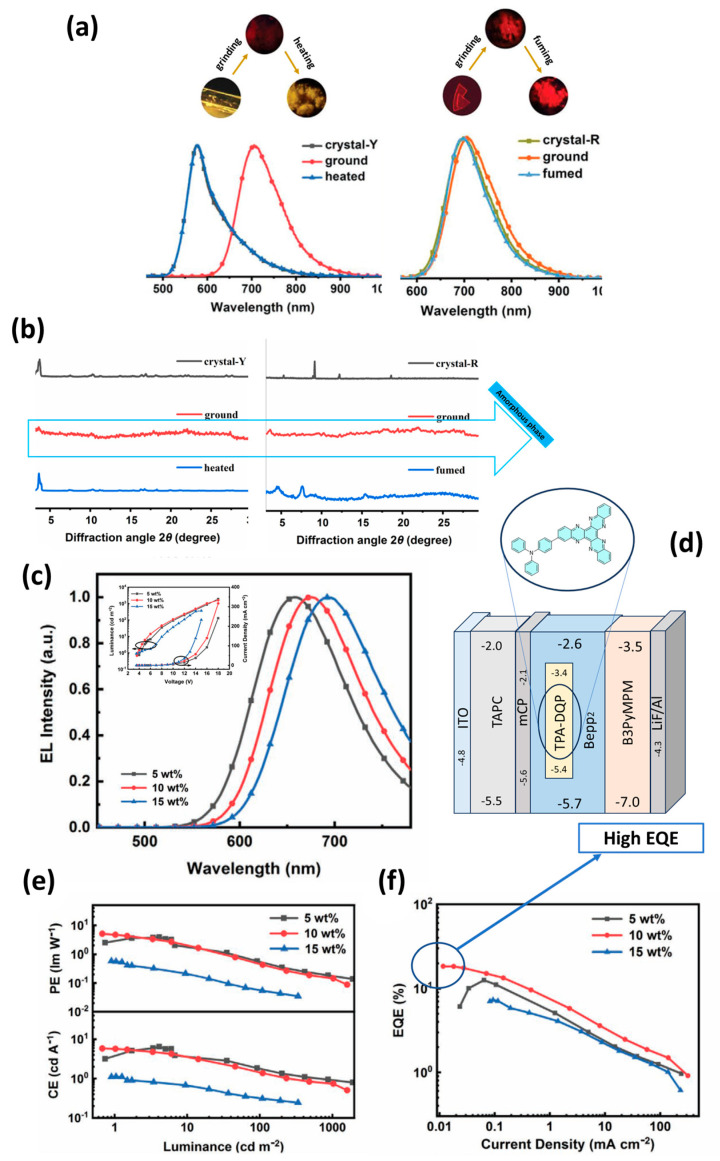
(**a**) Fluorescence photos and PL spectra of crystal Y and crystal-R in response to external stimuli. The photos were taken under UV irradiation (λ_exc_ = 365 nm). (**b**) PXRD patterns of crystal-Y, milled samples and heated samples, and crystal-R, milled samples and smoked samples, (**c**) Electroluminescence spectra (EL) recorded at 100 cd.m^−2^. Inset: (Current density–voltage–brightness (J–V–L) curves). (**d**) Molecular structure of TPA-DQP and Schematic representation of the OLED device architecture, (**e**) PE and CE as a function of luminance of TPA-DQP-based devices. (**f**) EQE curves as a function of current density. Reanalyzed and modified from data reported in [117].

Yu et al. developed a new multifunctional molecule, 4-(diquinoxalino [2,3-a:2′,3′-c]phenazin-2-yl)-N,N-diphenylaniline (TPA-DQP), by combining a triphenylamine (TPA) donor moiety with a large π-conjugated electron-deficient diquinoxalino [2,3-a:2′,3′-c]phenazine (DQP) acceptor (Figure 23d). A doped film of TPA-DQP in bis [2-(2-hydroxyphenyl)pyridinato]beryllium (Bepp₂) at a concentration of 10 wt% showed a maximum photoluminescence quantum yield (Φ_PL_) of 0.65 and a PL spectrum peak at 676 nm. TPA-DQP exhibited polymorphism, efficient TADF emission (with a low singlet–triplet energy splitting, ΔE_ST_, of 0.11 eV) and MCL behavior with high-contrast emission color changes from 576 nm to 706 nm. Two crystal forms of TPA-DQP, designated as crystal-Y (yellow, emitting at 576 nm) and crystal-R (red, emitting at 698 nm), were successfully prepared under different conditions. As shown in Figure 23a, heating the ground sample of crystal-Y in air at 240 °C converted it back to yellow microcrystals with emission maxima at 578 nm. Similarly, grinding crystal-R resulted in NIR emitting powders (emission peak at 706 nm). When the milled sample of crystal-R was exposed to CH₂Cl₂ vapor for 5 min, the emission increased again to 694 nm (Figure 23a). These results confirmed the mechanochromic properties of crystal-Y and crystal-R, which was also confirmed by the PXRD patterns (Figure 23b). To evaluate the electroluminescence (EL) of TPA-DQP as a TADF emitter, OLED devices with the following structure were fabricated: ITO/TAPC (40 nm)/mCP (5 nm)/EML (25 nm)/B3PyMPM (50 nm)/LiF (1 nm)/Al (100 nm), where the EML consists of 5, 10, and 15 wt% TPA-DQP in Bepp₂ (Figure 23d). The EL spectra, current density–voltage–luminance curves (J–V–L), power efficiency–luminance–current efficiency curves (PE–L–CE), and EQE–current density plots (EQE–J) are shown in Figure 23c,e,f. The best performing device doped with 10 wt% TPA-DQP achieved deep red emission (λ_em_ = 676 nm) with the following performance characteristics: Maximum CE: 5.69 cd/A, Maximum PE: 4.70 lm/W, Maximum EQE: 18.3% [117].

**Figure 24:**
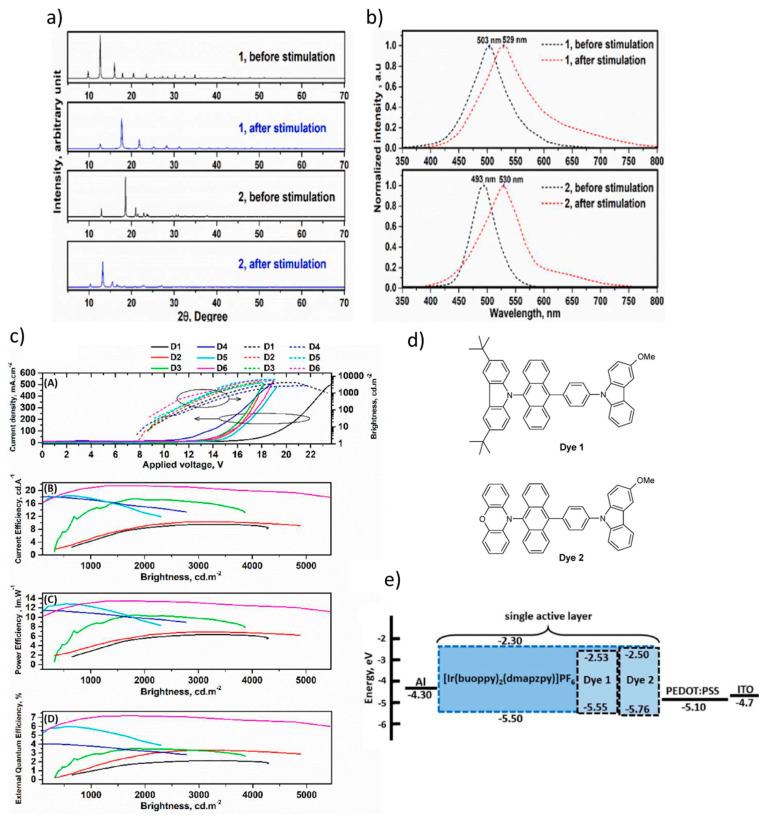
(**a**) Powder X-ray diffraction (XRD) patterns of before and after stimulation of dyes 1 and 2, and (**b**) PL spectrum of before and after stimulation of dyes 1 and 2. (**c**) (**A**) Applied voltage vs. brightness and current density, (**B**–**D**) current, power, and external quantum efficiency curves. (**d**) Molecular structure of dye 1 and dye 2. (**e**) Energy level of LEC structure [118].

Rabiei et al. reported, for the first time, the use of a mechanochromic TADF fish-shaped structured emitter in a LEC [118]. They designed and synthesized two TADF dyes, 5-(3,6-di-tert-butyl-9H-carbazol-9-yl)-10-((3-methoxy-9H-carbazol-9-yl)phenyl)anthracene (dye 1) and 5-(phenoxazine)-10-((3-methoxy-9H-carbazol-9-yl)phenyl)anthracene (dye 2), as emitters in the active layer of the LEC (Figure 24d). Dye 2 exhibited a lower ΔE_ST_ of 0.04 eV and showed mechanochromic behavior in the crystalline state. According to Figure 24b, mechanical stimulation induced a red-shift in the photoluminescence (PL) emission, shifting from 503 nm and 493 nm for pristine dyes 1 and 2, respectively, to 529 nm and 530 nm after grinding. This behavior indicates a significant change in molecular packing upon mechanical force. Powder X-ray diffraction (PXRD) analysis (Figure 24a) revealed sharp, well-defined peaks for the raw dyes, indicating high crystallinity. After mechanical stimulation, the intensity of the diffraction peaks decreased, the peaks broadened due to reduced crystallite size, and the sharpest peaks shifted to 2θ = 17.75° and 13.22°, confirming the structural change upon grinding. The LEC devices were fabricated with the architecture ITO/PEDOT:PSS (30 nm)/dye (80 nm)/Al (80 nm), where PEDOT:PSS served as both the HTL and anode buffer layer (Figure 24e). The best-performing device, utilizing dye 2 doped with 20 wt% [Ir(buoppyh(dmapzpy))]PF_6_ (D6), achieved a maximum CE of 21.40 cd/A, PE of 13.46 lm/W, and an EQE of 7.13% [118].

## 3. Conclusions

This review underscores the remarkable advances in LECs and OLEDs that have been driven by innovative material designs and device architectures. LECs offer unique advantages over OLEDs with their simplified manufacturing process, low-cost scalability and use of mobile ions, particularly in achieving low turn-on voltages and tunable emission characteristics. The use of TADF materials has significantly increased device efficiency by utilizing singlet and triplet excitons, achieving an IQE of nearly 100%. In addition, mechanochromic materials have emerged as versatile components with reversible optical changes upon mechanical excitation, enabling applications in sensing, structural monitoring and secure encryption technologies. The synergistic combination of TADF and mechanochromic properties has led to the development of multifunctional materials that exhibit powerful electroluminescence and reversible emission behavior under mechanical action. In particular, several case studies have demonstrated the successful implementation of D-A-D’ patterned dyes in OLEDs and LECs, achieving external quantum efficiencies above 14%, high photoluminescence quantum yields (up to 66%), and reversible mechanoresponsive color shifts confirmed by XRD and PL analyzes. These outcomes are summarized in Table 3, which provides a comparative overview of the key photophysical and device performance parameters of TADF and mechanochromic materials used in OLED and LEC devices. Despite these successes, challenges such as oxygen sensitivity, triplet quenching, limited emitter stability, and structural complexity of mechanochromic TADF materials remain. The performance of LECs is still limited by relatively slow response times and shorter operating lifetimes compared to OLEDs. Nevertheless, the potential of mechanochromic TADF emitters for solvent-based, flexible and portable optoelectronic devices remains high. By combining TADF and mechanochromic properties, multifunctional emitters have been developed that offer improved performance, including high efficiency, stability, and compatibility with solution processes for flexible electronics. Looking to the future, the synergistic integration of these advanced materials into LECs and OLEDs paves the way for next-generation lighting and display technologies. Future research will focus on overcoming challenges such as scalability, operational lifetime, and environmental stability, while expanding the scope of applications for wearable devices and large-scale commercial production. This progress underscores the potential of TADF and mechanochromic systems in bridging the gap between high efficiency, versatility, and sustainable manufacturing. Additionally, the integration of machine learning and multiphotonic effects presents a promising direction for future research in TADF and mechanochromic materials. ML can assist in predicting emission behaviors, improving structural design, and accelerating the development of high-performance optoelectronic devices.

## Figures and Tables

**Table 1:** Advantages and disadvantages of OLEDs and LECs.

Device	Advantages	Disadvantages
OLEDs	✓ High brightness and contrast ratio ✓ Fast response time ✓ Wide viewing angles ✓ Flexible and transparent display possibilities ✓ High color purity and tunability	○ Complex multilayer structure ○ Requires expensive vacuum deposition for fabrication ○ Shorter lifetime for blue emitters ○ Sensitive to moisture and oxygen ○ Higher production costs
LECs	✓ Simple device architecture (typically single-layer) ✓ Solution processable (suitable for printing) ✓ Lower fabrication costs ✓ Air-stable electrodes can be used ✓ Self-doping enables balanced charge injection	○ Slower response time ○ Lower brightness and efficiency compared to OLEDs ○ Limited operational lifetime ○ Less mature technology (limited commercial application) ○ Color tuning can be more challenging

**Table 2:** Comparison table: TADF and mechanochromic materials in OLEDs and LECs.

Device Type	Advantages	Disadvantages
TADF-based OLEDs and LECs	✓ High internal quantum efficiency (up to 100%) without heavy metals ✓ Potential for low-cost fabrication, especially in LECs ✓ Simplified device structure in LECs and high brightness in OLEDs ✓ Enhanced exciton utilization via RISC mechanism	○ TTA and stability issues at high brightness ○ Precise energy-level alignment required ○ In LECs, slower response time due to ionic motion Limited number of stable TADF emitters for LEC environment ○ Synthetic complexity and limited availability of suitable TADF emitters ○ Synthetic complexity and limited availability of suitable TADF emitters
Mechanochromic-based OLEDs and LECs	✓ Dual functionality: luminescence and stress-responsive color changes ✓ Useful for sensors, smart labels, and flexible devices ✓ Potential for low-cost and solution-processable fabrication	○ Generally lower luminescence efficiency ○ Mechanical fatigue or irreversibility over multiple cycles ○ Limited mechanochromic materials suitable for LEC operation ○ Requires specific molecular design for balanced performance ○ No possibility of identification through XRD testing before and after mechanical stress ○ Incompatibility with specific cathode materials (e.g., Al)

**Table 3:** Summary of photophysical characteristics and device performance parameters of reviewed TADF and mechanochromic materials used in OLED and LEC architectures.

Device/Configuration or Compound	λ_PL_ (nm)	CIE (X, Y)	V_on_ (V)	CE_max_ (cd/A)	PE_max_ (lm/W)	EQE_max_/%	Lmax (cd/m^2^)	τ_d_ /µs	ΔE_ST_/eV	λ_EL_ (nm)	Φ_PL_ /%	EQE_100_/%	EQE _1000_/%	Ref
OLED/D-A-D’ dye	475/518/472 initial/well grinded/stimulated	(0.148, 0.269)/ (0.322, 0.588)/(0.131, 0.208) initial/well grinded/stimulated (0.1899, 0.2593)	5.09 (at 63.68 cd/m^2^)	37.32	20.99	13.41	72,565	1.956	0.37	473	41	N/A	N/A	[96]
OLED/5,7TzPmPXZ	543	(0.43, 0.53)	N/A	41.9	N/A	14.03	12,210	2.6	0.06	560	66	13.5	12.4	[102]
OLED/TATP-BP (non-doped)	520	(0.38, 0.55)	2.7	18.9	19.2	6.0	N/A	0.91	0.129	541	24.2	roll-off of 3.3%	N/A	[105]
OLED/QBP-DMAC	508	(0.30, 0.53)	3.6	56.8	55.8	18.8	2264	1.87 ms	0.33	523	78	5.8	2.0	[106]
OLED/D-A/diphenyl pyrimidine b	566/483 initial/well grinded	(0.20,0.39)	3	34.2	29.8	14.2	7385	2.52	0.077	490	43	11.5	N/A	[107]
OLED/Qx-Py-2DMAC	550	(0.39, 0.54)	2.82	33.6	30.1	11.1	3336	3.18	0.06	548	94	N/A	5.1	[108]
OLED/DCNQ-DMAC	546	N/A	N/A	47.7	42.9	14.6	13,576	1.4	0.02	528	36	N/A	N/A	[109]
OLED/2BPy-mTC	456/495 initial/well grinded	(0.17, 0.33)	4.5	39.4	24.8	16.3	7266	31.8	0.2	484	42	N/A	N/A	[110]
OLED/DMAC-2FDPS	485	N/A	3.1	46.8	42.0	21.2	5239	2.0	0.03	488	93	16.5	10.4	[111]
OLED/mDCBP	460/510 initial/well grinded	(0.16; 0.25)	3.6	34.0	26.5	18.4	8900	0.2	0.06	474	90	N/A	N/A	[112]
OLED/MeTPA-BQ	650	(0.64, 0.34)	3.4	5.88	5.43	10.1	N/A	40.1	650	0.01	42	3.4	1.4	[113]
OLED/MTPA-BPSB	527	(0.30, 0.50)	3.2–4.8	61.6	37.2	20.5	N/A	10.6	0.07	527	76	N/A	N/A	[114]
OLED/XT-BDPDBA	486	(0.158,0.341)	3	55.0	57.5	27.0	19,319	2.4	0.025	484	91	N/A	22.0	[115]
OLED/4CzPTANMe	602	(0.59, 0.41)	2.74	15.9	18.2	5.96	N/A	0.28	0.03	538	40 (Φ_FL_)	N/A	N/A	[116]
OLED/TPA-DQP	676	(0.67, 0.32)	3.8	5.69	4.7	18.3	1597	107.1	0.11	676	65	N/A	N/A	[117]
LEC/fish-shaped structures based on carbazole derivatives	520	(0.344, 0.590)	8.72	21.10	13.46	7.13	5944	6.8	0.04	533	39	N/A	N/A	[118]

## Data Availability

No new data were created or analyzed in this study. Data sharing is not applicable to this article.
